# Unveiling ammonia-induced cell death: a new frontier in clear cell renal cell carcinoma prognosis

**DOI:** 10.3389/fimmu.2025.1636977

**Published:** 2025-07-31

**Authors:** Peize Yu, Qikai Zhong, Xinlei Wang, Yifang Liu, Qiang Liu, Yuqiang Zhang, Jiawei Lu, Yang Dong, Cong-hui Han

**Affiliations:** ^1^ Department of Urology, Xuzhou Medical University, Xuzhou, Jiangsu, China; ^2^ Department of Urology, Xuzhou Central Hospital, Xuzhou, Jiangsu, China; ^3^ Jiangsu Key Laboratory of New Drug Research and ClinicalPharmacy, Xuzhou Medical University, Xuzhou, Jiangsu, China; ^4^ Department of Central Laboratory, Engineering Research Center of Cancer Cell Therapy and Translational Medicine, Xuzhou, Jiangsu, China

**Keywords:** clear cell renal cell carcinoma, ammonia-induced cell death, prognostic risk model, *ATP1A1*, metabolic reprogramming, immune microenvironment

## Abstract

**Background:**

Clear cell renal cell carcinoma (KIRC) is the most aggressive renal carcinoma subtype of renal carcinoma, characterized by high mortality, early metastasis, and resistance to treatment. Ammonia-induced cell death (AICD) has recently been identified as a novel metabolic mechanism influencing tumor progression, yet its prognostic implication and regulatory networks in KIRC remain underexplored.

**Methods:**

Transcriptomic and clinical information from the TCGA-KIRC cohort and the validation cohort (E-MTAB-1980) were analyzed. Differentially expressed AICD-related genes were identified through differential expression analysis, univariate Cox regression, and machine learning algorithms (LASSO, random forest, and CoxBoost). A prognostic risk model was developed via multivariate Cox regression. Spatial and single-cell transcriptomics were employed to characterize the immune microenvironment heterogeneity. Cell-based experiments were performed to investigate the potential involvement of *ATP1A1* in KIRC. Molecular docking and pan-cancer analyses were conducted to identify therapeutic candidates and ATP1A1-related mechanisms.

**Results:**

Five AICD-related genes (*FOXM1*, *ANK3*, *ATP1A1*, *HADH*, and *PLG*) were identified and selected to construct a risk score model. The model demonstrated high accuracy and was integrated into a nomogram for clinical application. High-risk (HR) patients exhibited immunosuppressive microenvironments, elevated tumor mutational burden (TMB), and genomic instability. *In vitro* functional assays confirmed that *ATP1A1* knockdown significantly enhanced the proliferative, migratory, and invasive capabilities of renal carcinoma cells (A498 and 786-O), suggesting a suppressive role for *ATP1A1* in malignant tumor progression. *ATP1A1*, a core gene, was associated with metabolic reprogramming and chemotherapy sensitivity across multiple cancers. Molecular docking revealed Emodinanthrone as a high-affinity ligand for *ATP1A1* (−6.8 kcal/mol).

**Conclusion:**

This study identifies an AICD-associated gene signature as a robust prognostic tool for KIRC, revealing its interactions with immune evasion and genomic instability. *ATP1A1* is proposed as a promising therapeutic target, with Emodinanthrone emerging as a novel drug candidate. These findings contribute to the advancement of personalized treatment strategies for KIRC patients.

## 1 Introduction

Renal cell carcinoma (RCC) represents one of the most common malignancies of the urinary system and is associated with a mortality rate ranging from 30% to 40% (1, 2). Among its subtypes, clear cell renal cell carcinoma (KIRC) represents the most frequent and aggressive form, constituting 70–80% of RCC cases globally (3). Data from the American Cancer Society indicate that the incidence of KIRC continues to rise annually, with significant variability in patient prognosis; notably, a subset of patients develops metastatic disease even at early stages (4). Despite recent advancements in surgical techniques, targeted therapies, and immunotherapies, the 5-year survival rate for KIRC patients remains poor, particularly in advanced stages (5). Current treatment options for advanced metastatic RCC include T-cell checkpoint inhibitors and anti-angiogenic tyrosine kinase inhibitors; however, treatment efficacy remains limited due to considerable heterogeneity in drug tolerance and patient survival outcomes. Furthermore, the response rate to immunotherapy remains relatively low. Hence, there is an urgent need to identify novel biomarkers to improve the stratification and personalization of therapeutic strategies for KIRC patients.

The mechanisms of cell death are critically responsible for the initiation, progression, and treatment response of cancer (6). Cancer cells evade normal cell death pathways to facilitate uncontrolled proliferation and metastasis. Diverse forms of regulated cell death have been identified, including necroptosis, apoptosis, autophagy, ferroptosis, and others, each characterized by distinct the biological features and regulatory mechanisms (7–9). In recent years, novel modes of cell death, particularly those triggered by metabolic dysregulation, have emerged as a major focus of research. Ammonia, a key metabolic byproduct, plays a pivotal role in the pathogenesis, maintenance, and therapeutic responsiveness of multiple diseases (10). Studies have demonstrated that hyperammonemia can induce liver fibrosis and RIPK1-β-mediated cell death, closely associated with urea cycle dysfunction (11). In cancer, dysregulated ammonia metabolism is increasingly recognized as a novel mechanism contributing to cell death. For instance, glioblastoma cells release ammonia via glutamine metabolism, which activates the SREBP-1 signaling pathway, establishing a feedforward loop that enhances lipid synthesis and promotes therapeutic resistance. Inhibition of the glutamine transporter ASCT2 or glutaminase disrupts this loop, and in combination with the lysosomal inhibitor pimozide, induces mitochondrial oxidative stress, leading to tumor cell death (12). Moreover, research by Bo Huang and colleagues has shown that ammonia generated through mitochondrial glutamine catabolism accumulates within lysosomes, leading to lysosomal alkalinization and subsequent ammonia reflux into mitochondria. This cascade results in mitochondrial swelling, autophagy impairment, and T cell death (13). The elucidation of ammonia-induced cell death (AICD) offers a novel framework for understanding tumor cell fate. Nevertheless, although a strong association between ammonia metabolism and cell death regulatory pathways has been established, the precise molecular targets and the complete gene regulatory networks remain largely undefined. Particularly in KIRC, the expression profiles, dynamic regulatory mechanisms, and clinical prognostic implications of AICD-related genes have yet to be systematically characterized.

This study utilized transcriptomic data and clinical cohorts of KIRC from The Cancer Genome Atlas (TCGA) to systematically integrate differential expression analysis, univariate Cox regression models, and multi-dimensional machine learning algorithms (LASSO, Random Forest, and CoxBoost) for identifying AICD-associated signature genes and constructing a prognostic risk scoring model. The model’s predictive performance for overall survival was validated using an independent cohort, and further enhanced by incorporating clinicopathological parameters into a nomogram to refine the prognostic stratification framework. A comprehensive analysis of tumor immune microenvironment heterogeneity between risk subgroups was conducted, including evaluations of genomic instability and differential expression of immune checkpoint molecules. Functional validation through *in vitro* gene knockdown experiments revealed the regulatory roles of key genes in promoting malignant phenotypes. Molecular docking was employed to screen for potential therapeutic compounds and assess ligand–receptor binding affinities. Additionally, pan-cancer analysis explored multi-omics correlations between the target genes and metabolic reprogramming, immune cell infiltration, and chemotherapeutic sensitivity. Collectively, this study provides both theoretical insights and experimental evidence to support improved prognostic evaluation and the development of targeted therapies for KIRC.

## 2 Materials and methods

### 2.1 Cell culture

The human renal cell carcinoma cell lines A-498 and 786-O were purchased from Cyagen Biosciences (Guangzhou) Inc. and Seven Innovation (Beijing) Biotechnology Co., Ltd., respectively. All cell lines were cultured in RPMI 1640 medium supplemented with 100 U/mL penicillin, 100 μg/mL streptomycin, and 10% fetal bovine serum, in a humidified incubator at 37°C with 5% CO_2_.

### 2.2 Data collection

Overall, 1,120 AICD-related genes (DEARGS) were retrieved from GeneCards (https://www.genecards.org/) based on a relevance score >7 (14) (**Supplementary Table S1**). RNA-Seq expression data (HTSeq-FPKM format), along with respective clinical and survival data for KIRC patients, were obtained through UCSC Xena (http://xena.ucsc.edu/) (15). In total, 522 KIRC tumor specimens and 71 normal kidney tissue specimens were included for analysis. E-MTAB-1980, an independent validation cohort, was accessed via EMBL-EBI (https://www.ebi.ac.uk/) (16). To maintain uniformity in the data, ENSEMBL Gene IDs were transformed into Gene Symbol IDs, and genes exhibiting expression in <50% of the specimens were excluded. Additionally, the single-cell RNA sequencing (scRNA-seq) dataset GSE139555 (17) was downloaded from the GEO database, and KIRC spatial transcriptomic data were retrieved from GSE179572 (sample GSM5420752).

### 2.3 Human protein Atlas database analysis

HPA (http://www.proteinatlas.org) is a comprehensive and widely utilized resource for assessing protein levels across human cells and tissues, encompassing the Cell, Pathology, and Tissue Atlas (18). Immunohistochemistry (IHC) data for the genes included in the prognostic model were retrieved from the HPA database, specifically for renal cancer and adjacent normal tissues.

### 2.4 CCLE database analysis

The Cancer Cell Line Encyclopedia (CCLE) database (https://portals.broadinstitute.org/ccle) is a comprehensive resource containing data from hundreds of cancer cell lines. It provides valuable insights into various aspects of cancer biology, including molecular characteristics, drug responses, and genetic profiles. In this study, we analyzed the expression of *ATP1A1* across tumor cell lines using data from the CCLE database.

### 2.5 Processing of KIRC spatially annotated transcriptomic data

Spatial transcriptomic data were processed using the Seurat R package (19). The workflow included normalization of unique molecular identifier (UMI) counts, data scaling, and identification of highly diverse traits using the “SCTransform” function. Dimensionality reduction and unsupervised clustering were performed with “RunPCA,” utilizing the top 30 principal components based on significance. Default parameters were applied during clustering analyses. Subgroup and gene feature visualization were conducted using the “SpatialFeaturePlot” function. To assess AICD-related gene activity at spatial resolution, the AUCell R package (20) was employed, allowing quantification and visualization of localized gene expression signatures.

### 2.6 scRNA-seq data analysis

The 10× scRNA-seq data were transformed into a Seurat object using the Seurat R package. Quality control steps were implemented to filter out clusters containing fewer than three cells, cells expressing fewer than 50 genes, and cells in which mitochondrial gene expression exceeded 5% of total gene expression. Principal component analysis (PCA) was conducted based on the top 1,500 genes with the highest variability. Cell clustering was performed using the “FindClusters” and “FindNeighbors” functions, utilizing the top 15 principal components (PCs) for downstream analysis. Cluster-specific differentially expressed genes were determined using the “FindAllMarkers” function, applying a threshold of false-discovery-rate (FDR)<0.1 and |log_2_ fold-change (log_2_FC)|>1. Subsequent cluster annotation was performed by referencing the CellMarker 2.0 database (21) to accurately assign cell types. To quantify the activity of specific gene sets across individual cells, the ‘ssGSEA’ function integrated in the Seurat package was utilized.

### 2.7 Differential expression and functional enrichment analyses

Differentially expressed AICD-related genes (DEARGS) between KIRC and normal tissue specimens in the TCGA dataset were identified using the limma R package (22). Significance thresholds were set at FDR<0.5 and |log_2_ FC|>1. GO and KEGG pathway analyses were subsequently conducted using the Metascape platform (23), with an adjusted P<0.5 deemed statistically significant. Somatic mutation profiles of the identified DEARGS were analyzed and visualized using waterfall plots generated by the maftools R package (24). In addition, a PPI network of the DEARGS was constructed via STRING to explore potential functional interactions.

### 2.8 Development of the ammonia cell death scoring system

To identify the most prognostically relevant AICD genes, we applied a combination of LASSO regression, random forest analysis, CoxBoost regression, and stepwise Akaike information criterion (stepAIC) Cox regression analyses to the DEARGS. Genes with the strongest predictive value were subsequently incorporated into a multivariate Cox regression model. An ammonia cell death scoring system was constructed by linearly combining the regression coefficients from the multivariate Cox model, weighted by the normalized gene expression levels.


$$\text{Risk score}=\sum_{i=1}^{N}\left(Expi\times Coei\right)$$

Patients with KIRC were stratified into LR and HR cohorts in accordance with the median risk score derived from the scoring system. Overall survival (OS) differences between the two cohorts were assessed using Kaplan–Meier method and the log-rank test. To ensure the robustness and generalizability of the model, internal and external validation were subsequently performed using the TCGA and E-MTAB-1980 cohorts, respectively.

### 2.9 Application of a prognostic clinical model for KIRC

The construction of a nomogram is a widely utilized method for visualizing and implementing prognostic models in clinical practice. In this study, we performed both univariate/multivariate Cox regression analyses for identifying potential risk factors associated with KIRC prognosis, applying a significance threshold of P<0.5. These identified risk factors were subsequently incorporated into the development of a nomogram using the “rms” R package (25). The nomogram provides a graphical representation that allows for the estimation of 1-, 3-, and 5-year mortality probabilities based on cumulative points derived from the selected input variables. To assess the predictive performance of the nomogram, calibration curves for 1/3/5-year survival, as well as cumulative hazard curves, were generated and analyzed.

### 2.10 Assessment of immune characteristics

The relative abundances of 22 immune cell types were determined using the CIBERSORT R package (26). In addition, the expression profiles of immune checkpoint related genes were compared between risk groups to explore the potential for immunotherapy efficacy. The maftools R package was employed to generate waterfall plots, visualizing the pattern of genes with elevated somatic mutation frequencies in KIRC patients. Furthermore, the tumor mutation burden (TMB) for each specimen was measured to investigate the correlation between the risk score and TMB.

### 2.11 RNA interference

*ATP1A1*-targeting siRNAs (si*ATP1A1*#1, si*ATP1A1*#2, si*ATP1A1*#3, si*ATP1A1*#4) and a negative control siRNA were obtained from GenePharma. The siRNA sequences were as follows: si*ATP1A1*#1: Sense 5′-GCCGACUUGGUCAUCUGUATT-3′, Antisense 5′-UACAGAUGACCAAGUCGGCTT-3′; si*ATP1A1*#2: Sense 5′-CCGAGCAGCUGGAUGACAUTT-3′, Antisense 5′-AUGUCAUCCAGCUGCUCGGTT-3′; si*ATP1A1*#3: Sense 5′-CCAUCCAAUCACAGCUAAATT-3′, Antisense 5′-UUUAGCUGUGAUUGGAUGGTT-3′; si*ATP1A1*#4: Sense 5′-GCUGACCUCAGAAUCAUAUTT-3′, Antisense 5′-AUAUGAUUCUGAGGUCAGCTT-3′; siControl: Sense 5′-UUCUCCGAACGUGUCACGUTT-3′, Antisense 5′-ACGUGACACGUUCGGAGAATT-3′. Briefly, cells were seeded in 6-well plates and transiently transfected at 60–80% confluence using siRNA-Mate Plus transfection reagent (GenePharma) following the manufacturer’s instructions. For each well, 7.5 μL of transfection reagent was mixed with siRNA (at concentrations optimized through preliminary experiments) to form transfection complexes. After 12 hours of transfection, the medium was replaced with fresh culture medium. Cells were harvested 24 hours later for analysis of *ATP1A1* mRNA and protein levels by qPCR and western blotting, respectively.

### 2.12 Quantitative reverse transcription polymerase chain reaction analysis

qRT-PCR was used to measure *ATP1A1* mRNA expression levels. Total RNA was first extracted from the cells using RNA-easy Isolation Reagent (Vazyme, R701-01). The RNA was then reverse transcribed into cDNA following the protocol for HiScript II Q RT SuperMix for qPCR (+gDNA wiper) (Vazyme, R223-01). Finally, qPCR was performed using ChamQ SYBR qPCR Master Mix (Vazyme, Q711-02) to quantify *ATP1A1* mRNA levels, with *GAPDH* serving as the internal control. The primer sequences used were as follows: *ATP1A1* forward, 5’-GGATGACCGCTGGATCAACGATG-3’, *ATP1A1* reverse, 5’-GCACCACCACGATACTGACGAAG-3’, *GAPDH* forward, 5’-CAGGAGGCATTGCTGATGAT-3’, *GAPDH* reverse, 5’-GAAGGCTGGGGCTCATTT-3’.

### 2.13 Western blotting

A498 and 786-O cells were transfected with siRNA for 48 h, then harvested and lysed in RIPA buffer. Total protein (100 μg per sample) was separated by SDS-PAGE and transferred onto PVDF membranes (Millipore). After blocking with 3% non-fat milk in TBST, the membranes were incubated overnight at 4°C with primary antibodies: anti-ATP1A1 (1:5000, Proteintech, 14418-1-AP) and anti-β-actin (1:2000, Santa Cruz, sc-1616). Following this, HRP-conjugated secondary antibody (1:5000, ZSGB-BIO) was applied for 1 h at 37°C. Protein bands were detected using ECL reagent (Amersham Biosciences) and visualized with a chemiluminescence imaging system. β-actin was used as the loading control.

### 2.14 Cell proliferation

A498 and 786-O cells in the logarithmic growth phase (control and knockdown groups) were trypsinized, centrifuged, and counted. Cell suspensions were prepared at a concentration of 1×10^4^ cells/mL, and 100 μL was seeded into each well of 96-well plates (five replicates per group, across four plates). After 24 h of incubation, 10 μL of CCK-8 reagent diluted in 90 μL of fresh medium was added to each well under light-protected conditions. Following a 2-h incubation at 37°C, absorbance was measured at 450 nm using a microplate reader. This procedure was repeated every 24 h for a total of 96 h. Cell growth curves were plotted with time points (24, 48, 72, and 96 h) on the x-axis and normalized OD values on the y-axis. Three independent experiments were conducted. Data are presented as mean ± standard deviation (SD). Statistical comparisons among multiple groups were performed using one-way ANOVA followed by Dunnett’s *post hoc* test. A p-value < 0.05 was considered statistically significant.

### 2.15 Cell invasion assays

The invasive capacity of transfected cells was assessed using Transwell chambers (24-well format, 8.0 μm pore size; Corning, #3422). For the invasion assays, chambers were precoated with Matrigel (Corning, #354234). Transfected cells (1.0 × 10^5^) suspended in 150 μL of serum-free medium were seeded into the upper chambers, while 600 μL of complete medium containing 10% FBS was added to the lower chambers. After 24 h of incubation, the invaded cells were fixed with methanol and stained with 0.1% crystal violet for 10 minutes at room temperature. Cells in five randomly selected fields per chamber (at 200× magnification) were counted.

### 2.16 Wound healing assay

A-498 and 786-O cells were seeded evenly into six-well plates. Once a confluent monolayer was formed, a straight scratch was made using a 200 μL pipette tip to create a uniform wound. Detached cells and debris were removed by gentle washing, and serum-free medium was added. Plates were then incubated at 37°C. Scratch width was measured, and images were captured at 0 and 48 hours using an inverted microscope. The experiment was repeated three times to evaluate the initial wound width and the extent of cell migration.

### 2.17 Molecular docking

The structures of the bioactive components were sourced from the Traditional Chinese Medicine Active Compound Library and imported into ChemBio3D v14.0 for spatial conformation adjustment, energy optimization, and export in the mol2 format. After processing with AutoDock Tools 1.5.6, the 3D crystal structures of target proteins were retrieved from UniProt. Water molecules and bound organic molecules were eliminated using Notedad2, and target proteins were subsequently prepared by adding hydrogen atoms, assigning charge distributions, and determining atomic types using AutoDock Tools 1.5.6. AutoDock Vina was employed for molecular docking, and the resulting docking poses were visualized and analyzed using PyMOL 2.6.1.

### 2.18 Pan-cancer analysis

The TCGAplot R package (27) was utilized to examine the relationships between *ATP1A1* expression levels among diverse cancer types in a pan-cancer context. Pearson correlation analysis was carried out to assess the statistical associations between *ATP1A1* expression and established immunotherapy biomarkers, such as microsatellite instability (MSI), immune cell infiltration, and other immune-associated genes among diverse cancer types. The mutation landscape of *ATP1A1* in various cancers was determined through cBioPortal (http://www.cbioportal.org/). Additionally, the cor.test R package was applied to evaluate the Spearman correlation between *ATP1A1* expression and the sensitivity to different chemotherapy drugs, based on data from the PRISM, GDSC, and CTRP databases. Methylation data was retrieved from the TCGA database, and bubble plots were generated to visualize variations in the methylation levels of *ATP1A1* at different loci across multiple cancers.

### 2.19 Statistical analyses

All statistical tests were performed using R software (v4.3.1). The Wilcoxon rank-sum test was applied for two-group comparisons, whereas the Kruskal-Wallis test was used for comparisons involving more than two groups. Survival outcomes were evaluated using Kaplan-Meier analysis, with differences assessed via the log-rank test. P<0.05 was indicative of statistical significance.

## 3 Results

### 3.1 Variant landscape of AICD genes in KIRC patients

**Figure 1:**
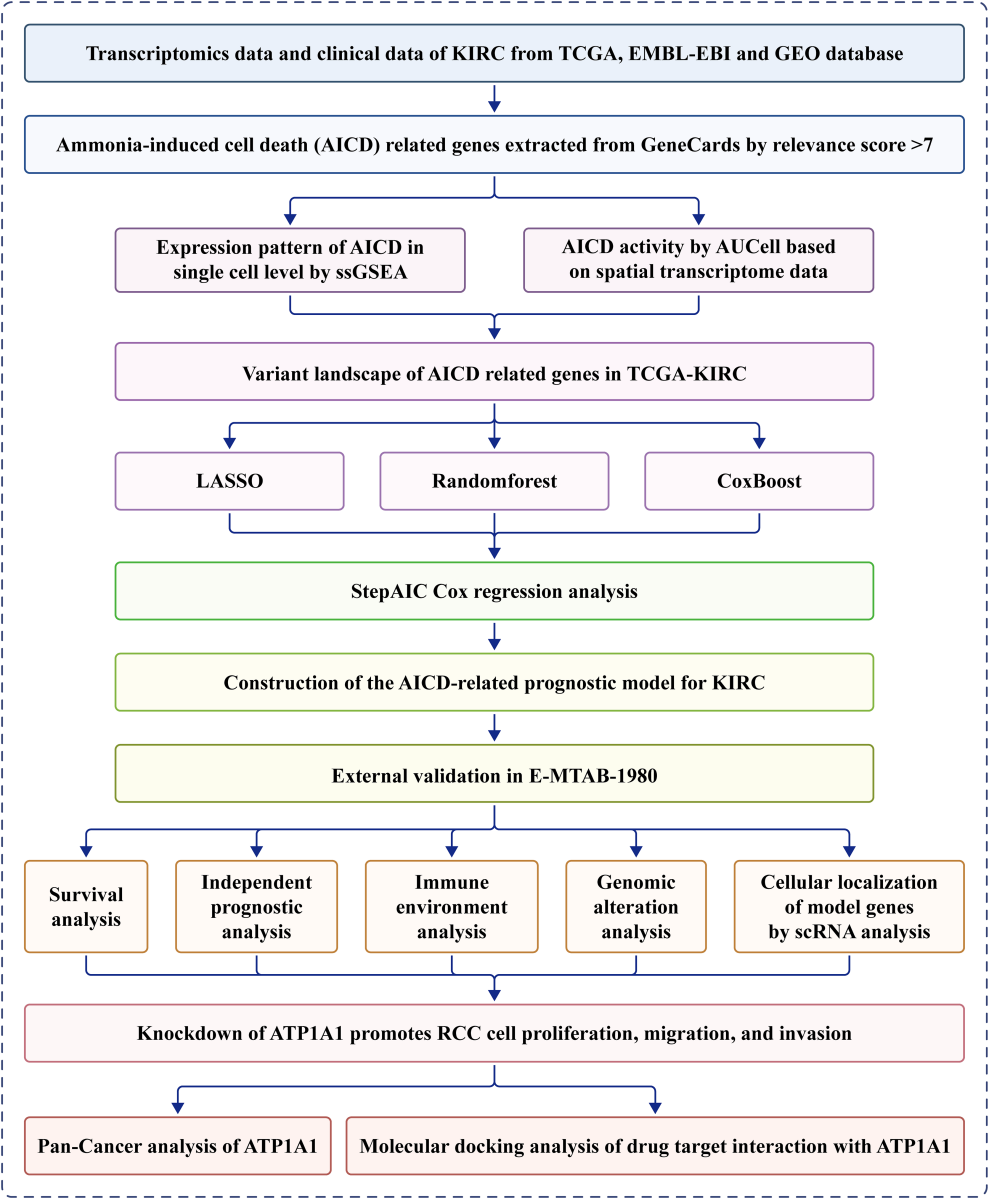
Flowchart of this study.

**Figure 2:**
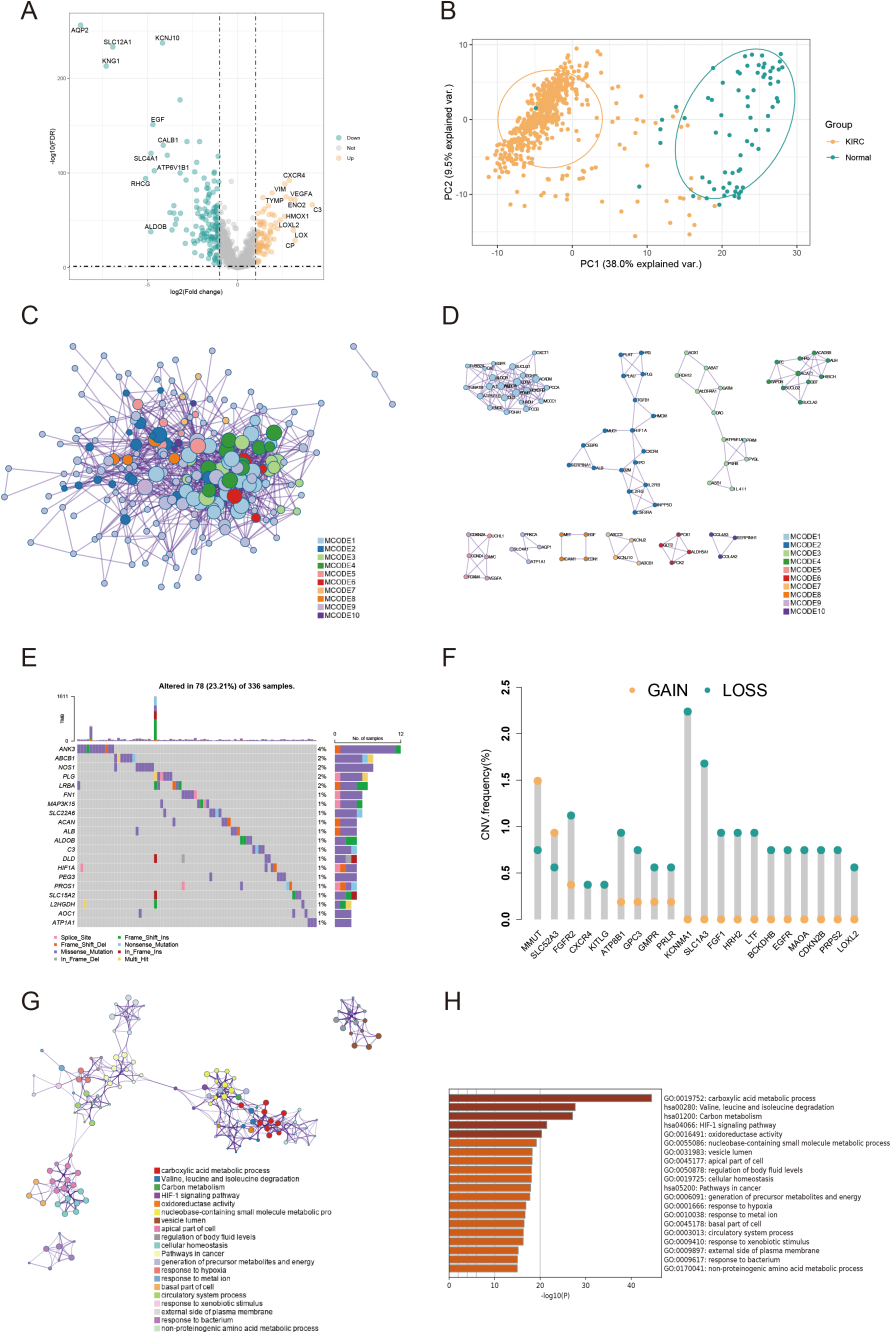
Variant landscape and functional characterization of AICD genes in KIRC patients. **(A)** Volcano plot depicting differentially expressed AICD-related genes (DEARGs) in KIRC (green: down-regulated DEARGS; yellow: up-regulated DEARGS; grey: unaltered genes), with FDR<0.5 and |log_2_FC|>1. **(B)** PCA showing obvious differences between KIRC and normal samples. **(C)** PPI network of AICD-related DEARGS constructed based on the Metascape database. **(D)** Identification of significant subnetworks through the MCODE algorithm. **(E)** Oncoplot of the top 20 AICD-related DEARGs in the TCGA cohort. **(F)** Rates of CNV loss, gain, and no CNV among the top 20 AICD-related DEARGS. **(G, H)** Functional enrichment analyses of AICD-related DEARGS.

A schematic overview of the study design is depicted in **Figure 1**. Overall, 228 DEARGs were identified between KIRC and normal specimens from the TCGA database (**Figure 2A**). The expression levels of each of the 228 DEARGs in the integrated dataset are depicted in **Figure 2B**. Next, a PPI network was established through Metascape, and critical modules within the network were analyzed using the MCODE plugin. Ten significant modules were identified, as illustrated in **Figures 2C, D**. Furthermore, the molecular alteration landscape of AICD-related DEARGs in KIRC was assessed, with missense mutations being the most frequent variant type, as shown in **Figure 2E**. Among these, the gene *ANK3* exhibited the highest mutation frequency. The analysis of CNV mutations revealed the top 20 DEARGs with the most obvious CNV alterations (**Figure 2F**). GO and KEGG enrichment analyses demonstrated that the DEARGs are primarily involved in processes such as carboxylic acid metabolism, the HIF-1 signaling pathway, oxidoreductase activity, response to xenobiotic stimulus, response to hypoxia, cellular homeostasis, and carbon metabolism (**Figures 2G, H**).

### 3.2 Immunogenic AICD characteristic in spatial and single-cell transcriptomics

**Figure 3:**
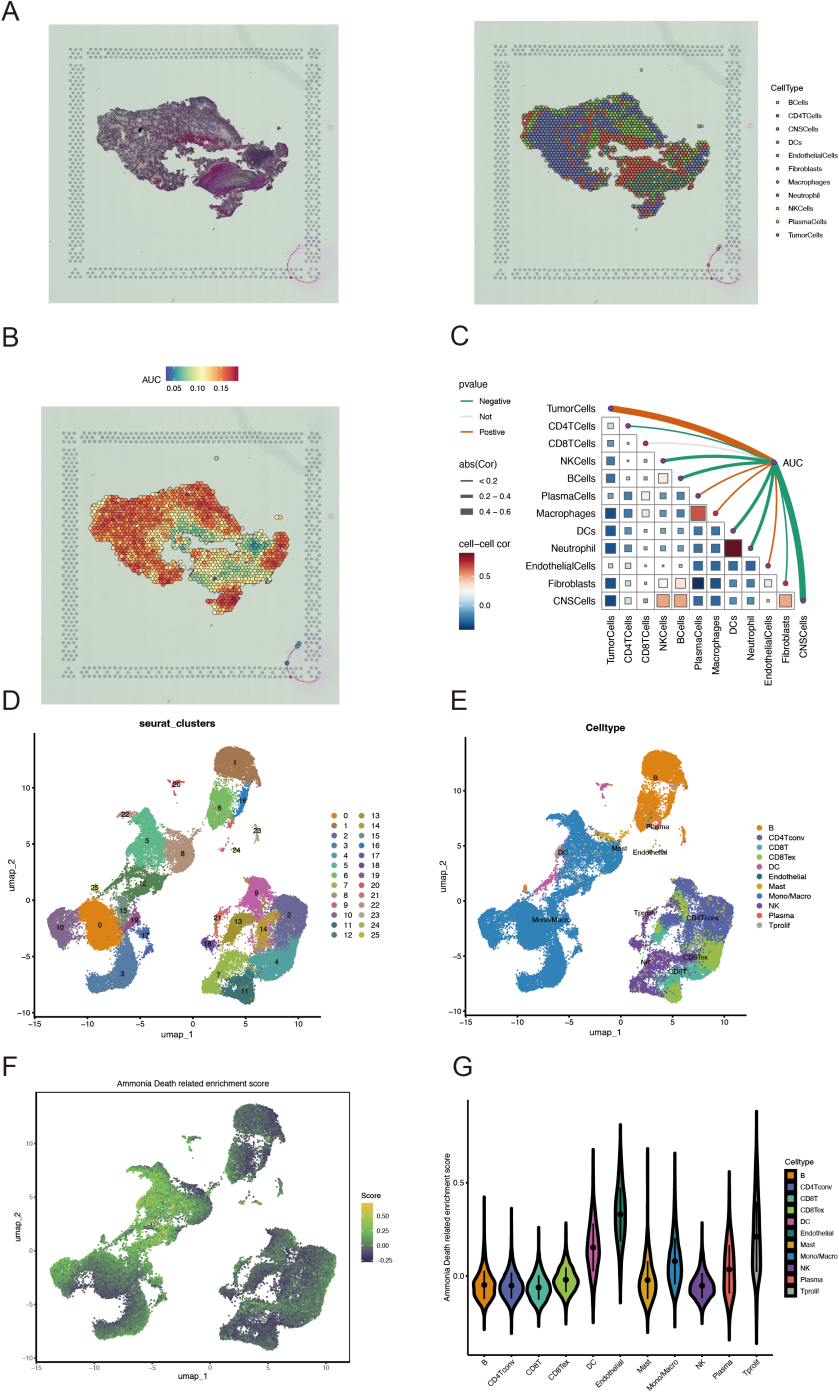
Characterization of AICD activity in spatial transcriptomics and single-cell RNA sequencing. **(A)** Spatial transcriptomics data of KIRC. **(B)** Spatial visualization of AICD intensity. **(C)** Spearman correlation assessment of the spatial profiles of AICD activity within the tumor microenvironment. **(D, E)** Identification of single cell types using marker genes. **(F)** AICD-related enrichment scores. **(G)** Distribution of AICD activity across different cell types.

We applied the SCTransform method to adjust for variations in spatial sequencing depth, followed by normalization procedures, which ultimately led to the identification of 11 distinct cell types through dimensionality reduction and clustering (**Figure 3A**). To assess the role of AICD-related DEARGs in each cell subset, we utilized the AUCell R package to evaluate AICD activity within each subgroup (**Figure 3B**). Subsequently, Spearman correlation analysis was conducted to examine the relationship between cell content and AICD activity across all spatial spots (**Figure 3C**). We obtained scRNA-seq data from 49,899 cells derived from three ccRCC patients. To eliminate batch effects, we utilized the Harmony package, achieving successful integration of the three samples. Dimensionality reduction was then conducted using PCA and UMAP based on the top 2,000 most variable genes. Clustering of the cells resulted in 26 distinct clusters at a resolution of 1.5 (**Figure 3D**). Cells were subsequently annotated into 11 major cell types based on marker gene expression: B cells, CD4+ T conventional cells (CD4Tconv), CD8+ T cells (CD8T), exhausted CD8+ T cells (CD8Tex), mast cells, endothelial cells, dendritic cells, plasma cells, NK cells, monocytes/macrophages, and proliferating T cells (Tprolif) (**Figure 3E**). To determine the immunogenic activities of AICD across these cell types, the ssGSEA function within the Seurat package was employed to compute expression scores for AICD-related DEARGs for each cell (**Figure 3F**). Among the 11 cell types, endothelial cells exhibited significantly higher AICD activity (**Figure 3G**).

### 3.3 Establishment of an AICD-related prognostic model

**Figure 4:**
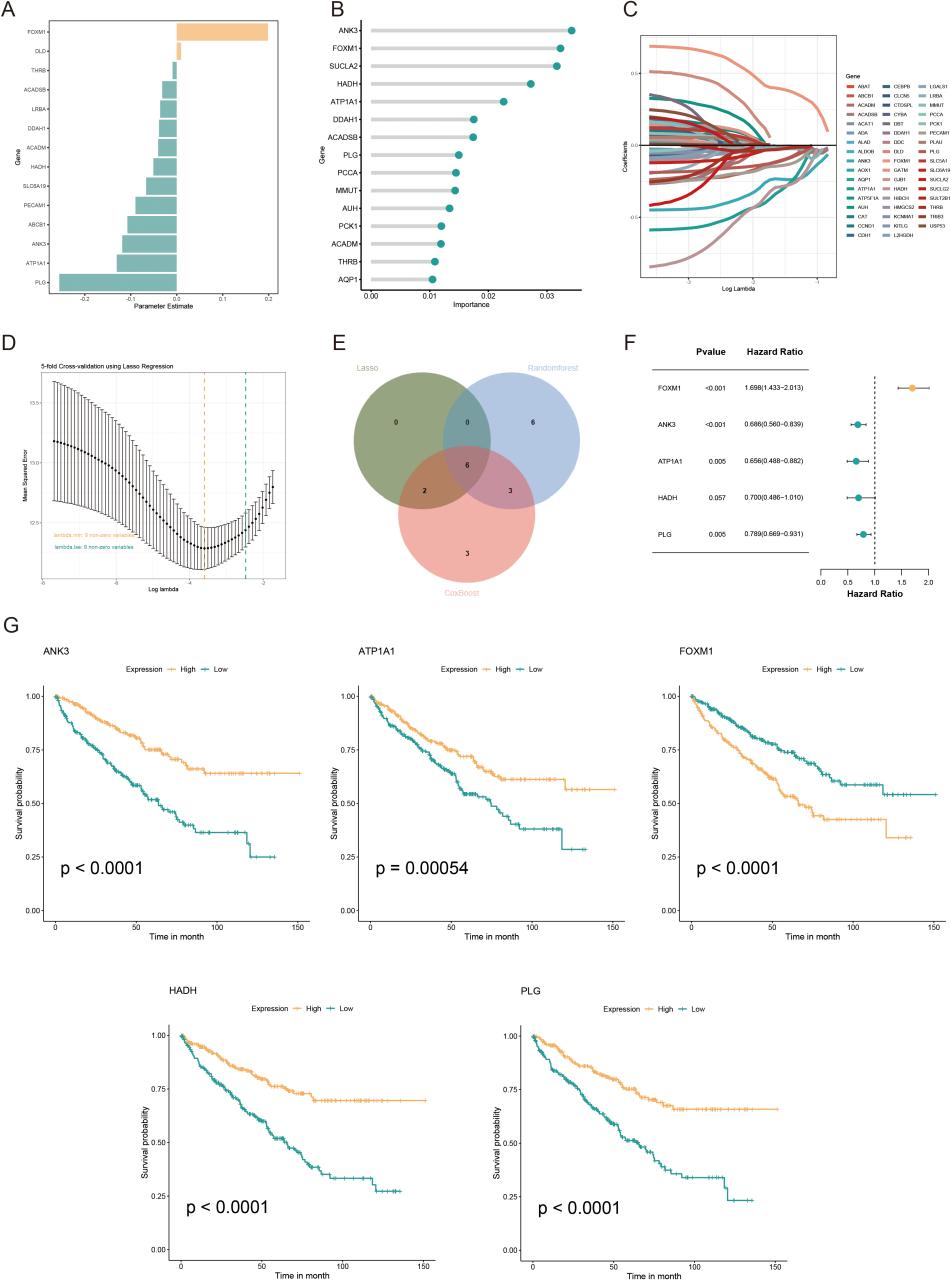
Development and validation of an AICD-associated prognostic signature for KIRC patients. **(A)** Feature selection according to the CoxBoost algorithm. **(B)** Identification of optimal biomarkers using the Random Forest (RF) algorithm. **(C, D)** Variable selection in the LASSO-Cox regression model. **(E)** Venn diagram indicating overlapping identified by the three algorithms. **(F)** Forest plots of the final 5 prognostic genes selected through stepAIC regression analysis. **(G)** Survival analysis of the prognostic genes in the TCGA-KIRC cohort.

**Figure 5:**
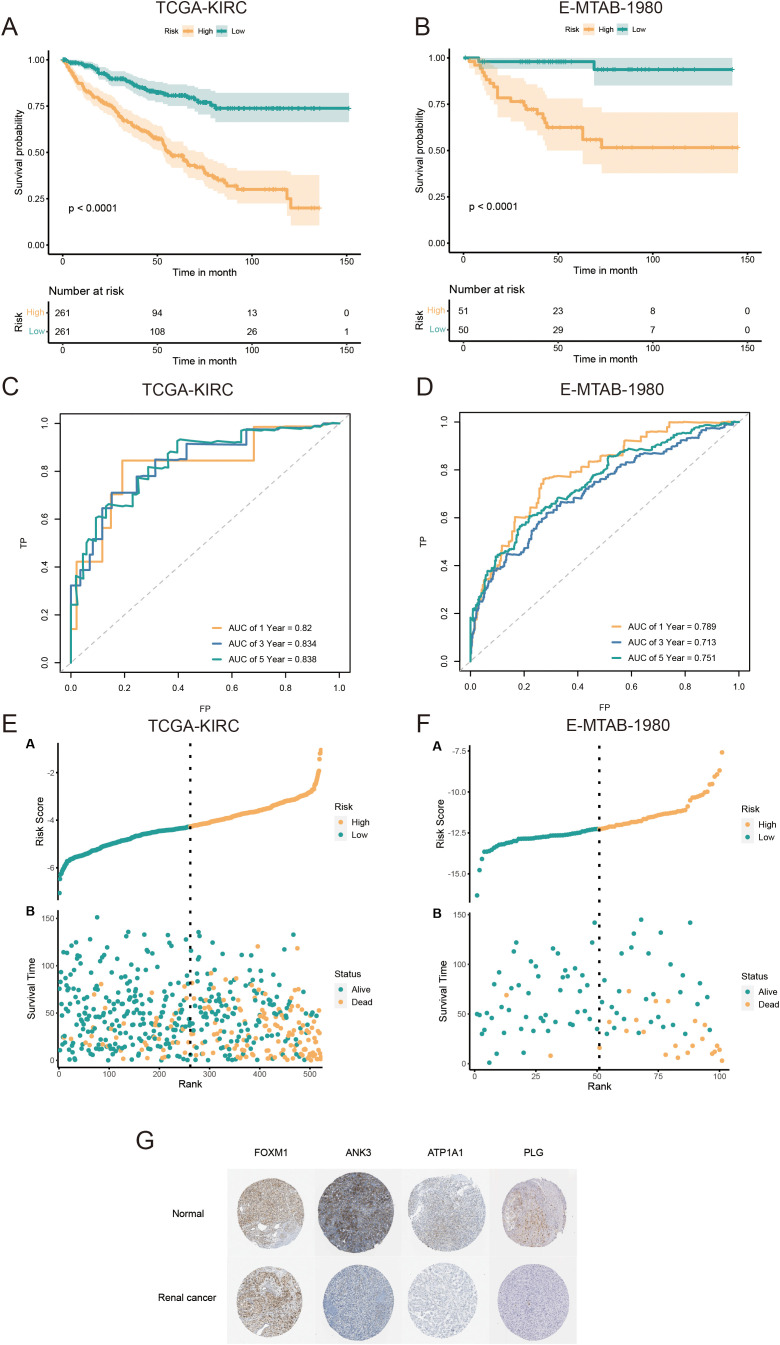
Validation and prognostic performance of the AICD-related signature in KIRC patients. OS of LR and HR patients in the TCGA-KIRC **(A)** and E-MTAB-1980 **(B)** cohorts. ROC curves of the prognostic model for estimating survival in the TCGA-KIRC **(C)** and E-MTAB-1980 **(D)** cohorts. (Risk score distribution stratified by survival status and time in the TCGA-KIRC **(E)** and E-MTAB-1980 **(F)** cohorts. **(G)** Expression of core prognostic model genes in renal cancer and normal tissues via the HPA database.

Univariate Cox regression analysis was used to identify 71 AICD-related genes from the TCGA-KIRC cohort. These genes were further evaluated through Kaplan-Meier survival analysis, leading to the identification of 51 genes with significant prognostic value. To mitigate overfitting risks and refine the selection of candidate biomarkers, three machine learning algorithms were employed to identify KIRC biomarkers with diagnostic significance. The CoxBoost algorithm identified 14 genes (**Figure 4A**), the Random Forest model identified 15 genes (**Figure 4B**), and LASSO regression analysis yielded 8 genes (**Figures 4C, D**). A Venn diagram was used to intersect these genes, revealing 6 robust core biomarkers: *FOXM1*, *ANK3*, *ATP1A1*, *HADH*, *THRB*, and *PLG* (**Figure 4E**). Subsequently, five genes, *FOXM1*, *ANK3*, *ATP1A1*, *HADH*, and *PLG*, were retained to construct an AICD-associated prognostic model via stepAIC Cox regression analysis (**Figures 4F, G**). The following formula was used to calculate the risk score for each patient: RiskScore = (*FOXM1* expression×0.5297) + (*ANK3* expression×−0.3772) + (*ATP1A1* expression×−0.4214) + (*HADH* expression×−0.3560) + (*PLG* expression×−0.2366). Based on the median risk score, patients were divided into LR and HR groups. Survival analysis indicated that patients in the LR group had a significantly better OS than those in the HR group, both in the TCGA-KIRC (**Figure 5A**, p<0.0001) and E-MTAB-1980 (**Figure 5B**, p<0.0001) cohorts. Additionally, the AUC for 1/3/5-year survival was assessed in both cohorts, revealing that the risk score provided high accuracy in estimating survival outcomes (**Figures 5C, D**). The risk scores and survival status distribution for both cohorts are presented in **Figures 5E, F**. These results confirm the robustness of the AICD-associated prognostic model in estimating the prognostic outcomes of KIRC patients. Furthermore, IHC analysis from the HPA database showed that the protein levels of the core genes aligned with the prognostic profile, validating the clinical relevance of the model (**Figure 5G**).

### 3.4 Development and evaluation of the nomogram survival model

**Figure 6:**
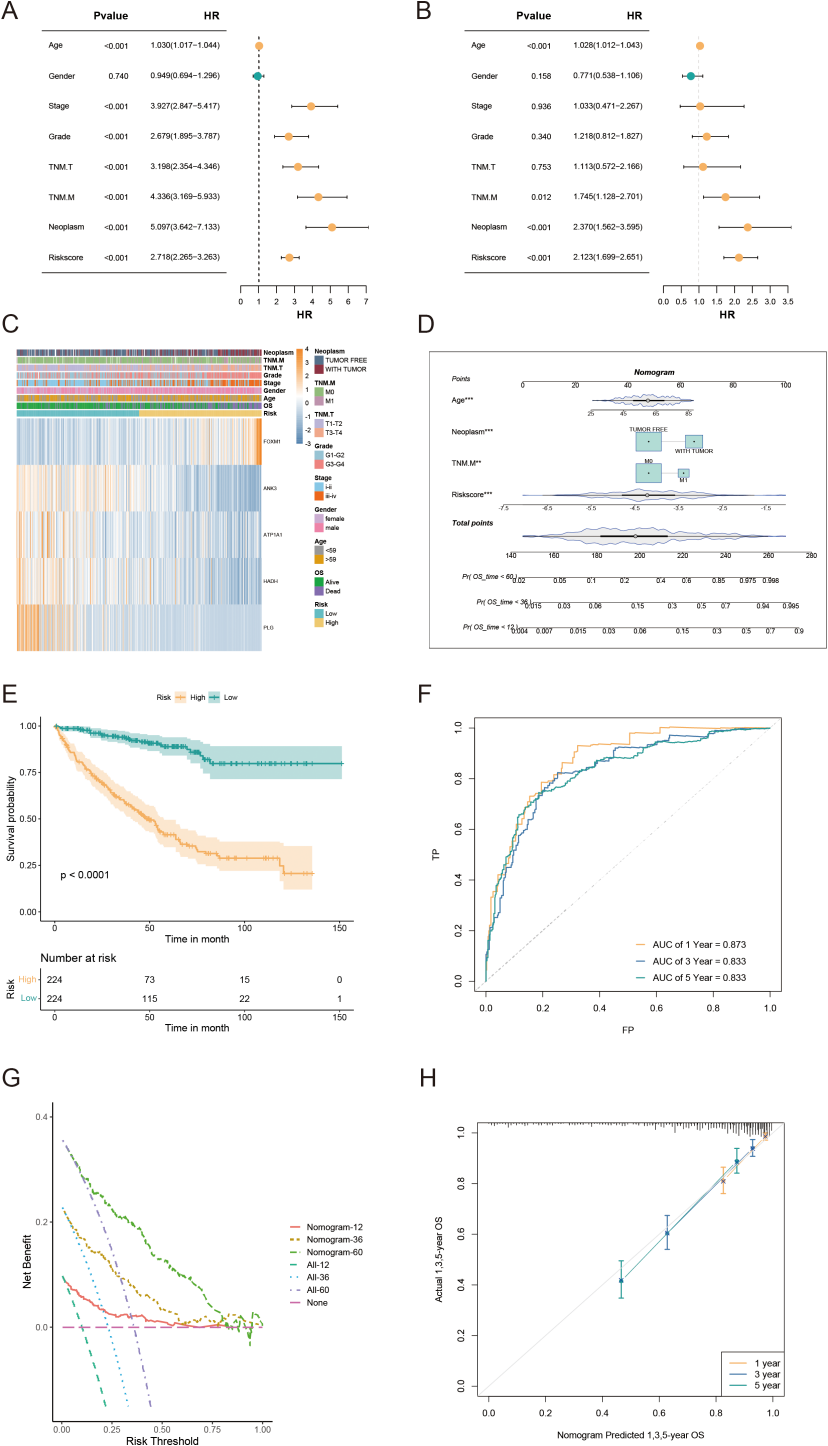
Nomogram development and evaluation for prognostic prediction in KIRC patients. **(A, B)** Univariate/multivariate Cox analyses of clinicopathologic traits and risk score in the TCGA-KIRC cohort. **(C)** Distribution of clinical characteristics and expression of model genes based on the AICD-related risk score. **(D)** Nomogram for predicting the prognosis of KIRC patients. **(E)** Kaplan-Meier survival analysis comparing LR and HR groups based on the nomogram score. **(F)** ROC curve analysis of the nomogram in the TCGA-KIRC cohort. **(G)** Calibration plots for predicting 1-, 3-, and 5-year overall survival in TCGA-KIRC. **(H)** DCA showing the net benefits of the nomogram compared to other clinical characteristics.

Univariate/multivariate Cox regression analyses revealed that the risk score was an independent prognostic factor for KIRC patients relative to other common clinical characteristics (**Figures 6A, B**). In the TCGA-KIRC cohort, both univariate/multivariate Cox analyses identified risk score, TNM.M, neoplasm status, and age as independent prognostic factors. The gene expression profiles, risk scores, and clinical factors for KIRC patients in the TCGA-KIRC cohort are visualized in **Figure 6C**. To evaluate the clinical applicability of the risk model, variables such as TNM stage, neoplasm status, and age were integrated into a nomogram developed to predict overall survival in KIRC patients from the TCGA-KIRC cohort (**Figure 6D**). The nomogram model demonstrated excellent prognostic performance compared to the gene signature model, with a significant prognostic difference between the LR and HR groups (P<0.001, **Figure 6E**). Additionally, the AUC for the combined model estimating 1/3/5-year OS was 0.873, 0.833, and 0.833, respectively (**Figure 6F**). Calibration curves further confirmed the accuracy of this model in estimating 1/3/5-year OS (**Figure 6G**). Furthermore, decision curve analysis (DCA) demonstrated that the nomogram model provided superior predictive performance compared to all other evaluated predictors (**Figure 6H**). Overall, the developed nomogram exhibits strong predictive power and clinical applicability, providing an effective tool for assessing the prognostic outcomes of KIRC patients based on key clinical indicators.

### 3.5 Correlation between immune microenvironment, immune characteristics, and the AICD-related prognostic model

**Figure 7:**
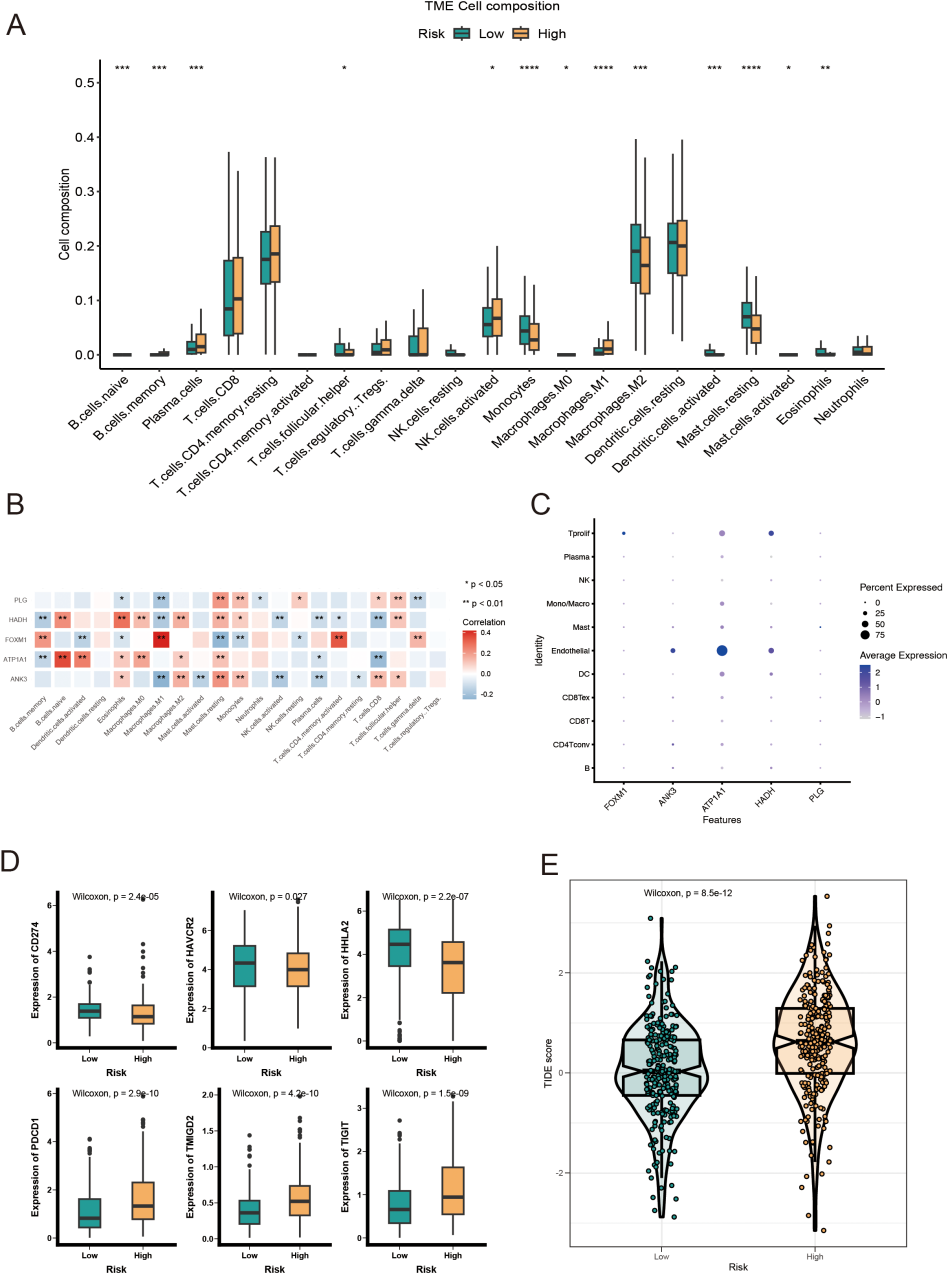
Immune landscape analysis of LR and HR KIRC patients based on AICD-related prognostic model. **(A)** Boxplot showing the abundance of 22 infiltrating immune cell types computed via CIBERSORT. **(B)** Correlation between TME infiltrating immune cells and genes in the AICD-related prognostic model. **(C)** Bubble plot indicating the average and percentage expression of prognostic biomarkers among diverse cell subtypes. **(D)** Boxplot illustrating the expression levels of IC-associated genes. **(E)** Violin plot of TIDE scores across risk groups. *p<0.5; **p<0.1; ***p<0.01; ****p<0.001.

To examine the immune infiltration landscape in ccRCC specimens, we quantified the abundance of tumor-infiltrating immune cells via the CIBERSORT algorithm (**Figure 7A**). Our analysis revealed that the LR cohort exhibited higher infiltration of T follicular helper cells, monocytes, M2 macrophages, and activated dendritic cells, while the HR cohort had elevated levels of activated NK cells, M1 macrophages, and plasma cells. In addition, we identified significant correlations between the prognostic model genes and immune cell infiltration. Specifically, *FOXM1* had a positive correlation with M1 macrophages and activated CD4+ memory T cells, while *ATP1A1* showed a positive correlation with naive B cells (**Figure 7B**). To further explore the expression of model genes in ccRCC patients, we analyzed scRNA-seq data (GSE139555). The dot plot analysis indicated that *ATP1A1* was predominantly expressed in endothelial cells (**Figure 7C**). Given the increasing clinical use of IC inhibitors (ICIs) as a form of immunotherapy, we examined the expression of IC-related genes between the LR and HR cohorts. The results showed significantly higher expression levels of *HAVCR2*, *CD274*, and *HHLA2* in the LR group, suggesting a differential susceptibility to IC inhibition (**Figure 7D**). In addition, we assessed the TIDE (Tumor Immune Dysfunction and Exclusion) score for each ccRCC patient and observed that the HR cohort exhibited a significantly higher TIDE score, suggesting a potential resistance to immunotherapy in these patients (**Figure 7E**). These data imply that ccRCC patients with an HR score may not benefit as much from immunotherapeutic strategies.

### 3.6 Genomic alterations in LR and HR KIRC patients based on AICD prognostic score

**Figure 8:**
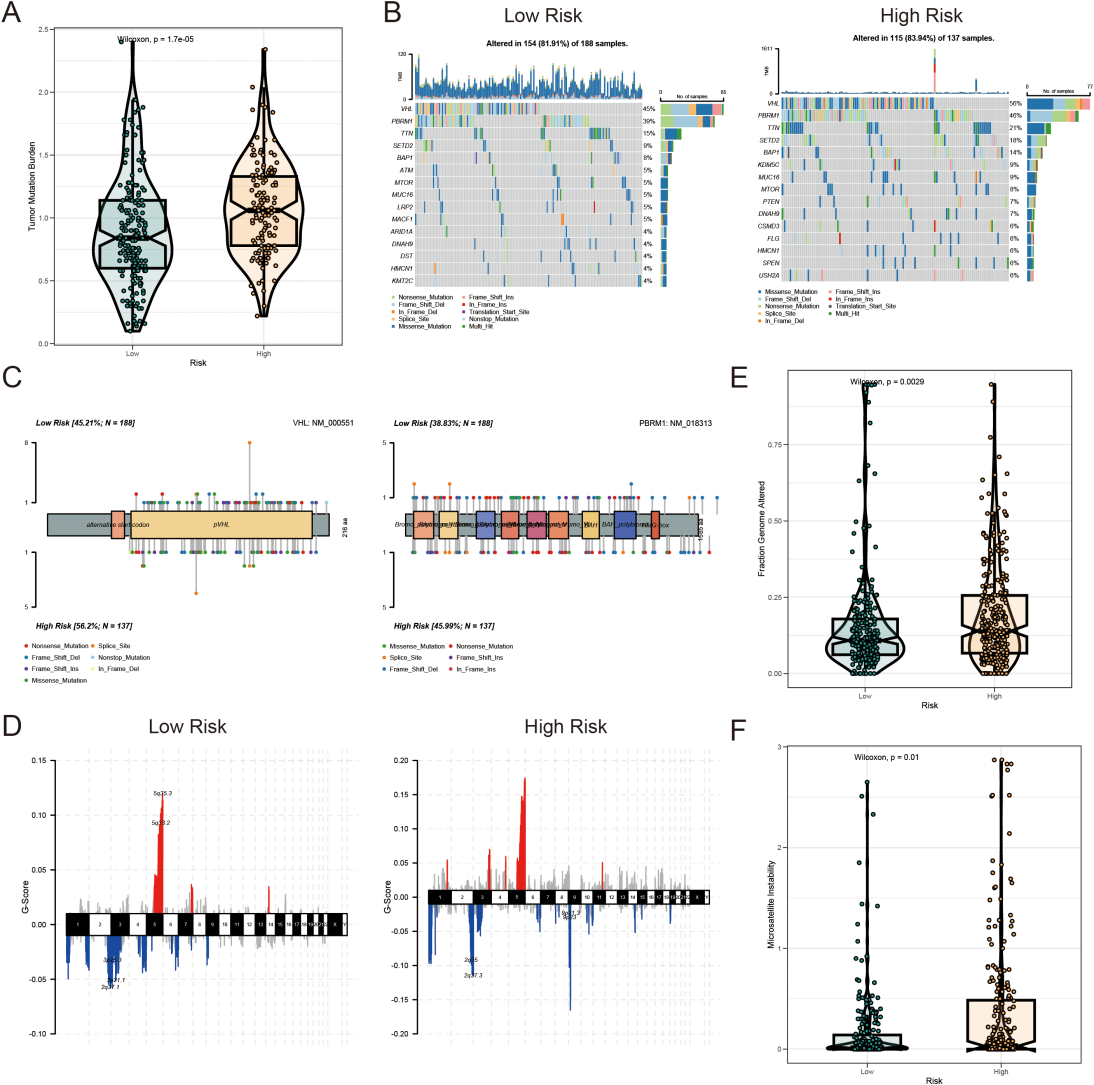
Mutation landscape of AICD-related prognostic subgroups in KIRC. **(A)** TMB analysis. **(B)** Waterfall plot depicting somatic mutation characteristics in HR and LR groups. **(C)** Comparison of several mutation loci in VHL and PBRM1 between risk groups. **(D)** CNV patterns in LR and HR groups. **(E)** FGA differences between risk groups. **(F)** Comparison of MSI across risk score categories.

HR KIRC patients exhibited a significantly higher TMB in protein-coding regions compared to LR patients (**Figure 8A**). The top 20 most frequently mutated genes were analyzed in both risk groups (**Figure 8B**). Notably, mutations in *VHL* (56% in HR vs. 45% in LR) and *PBRM1* (46% in HR vs. 39% in LR) were frequently observed in the HR group (**Figure 8C**). Copy number variation (CNV) analysis revealed distinct CNV patterns between the LR and HR AICD groups (**Figure 8D**), with the fraction of genome altered (FGA) significantly higher in the HR group (**Figure 8E**). These mutations, particularly those located in the DNA-binding domains of relevant proteins, may contribute to the compromised tumor-suppressive efficacy and reduced survival outcomes in patients. Furthermore, a high AICD risk score was associated with elevated microsatellite instability (MSI) status, which is recognized as a predictive marker for IC inhibition therapy (**Figure 8F**).

### 3.7 *ATP1A1* knockdown promotes proliferation, migration, and invasion in renal cell carcinoma cells

**Figure 9:**
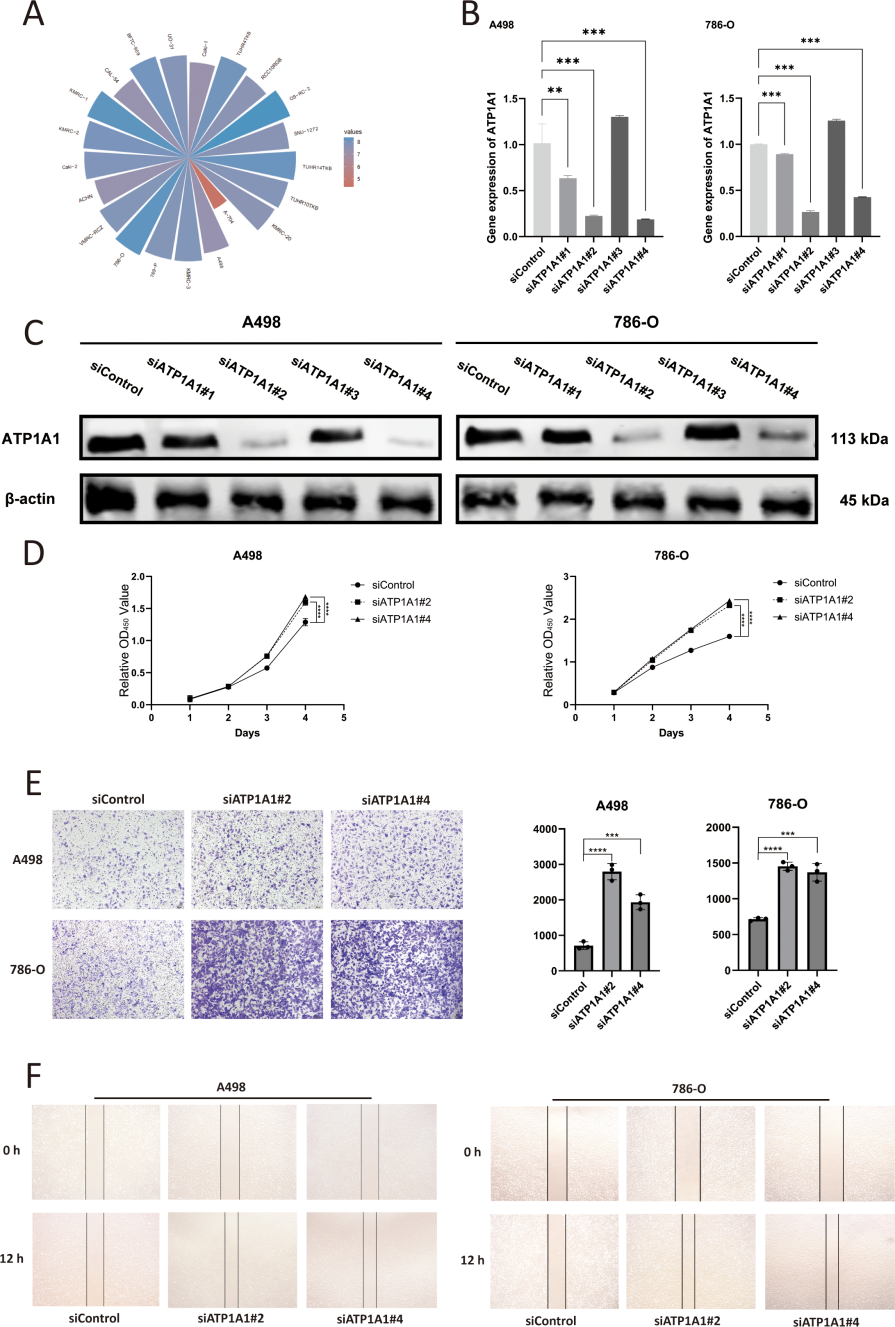
The effect of *ATP1A1* gene knockdown on the malignant biological functions of RCC cells. **(A)** *ATP1A1* expression across RCC cell lines based on CCLE data. **(B)** mRNA level of *ATP1A1* after si*ATP1A1* transfection. **(C)** Western blot analyses confirm the efficiency of *ATP1A1* knockdown in A498 and 786-O cells. **(D)** Cell proliferation after *ATP1A1* knockdown. **(E)** A498 and 786-O cells transwell invasion image after *ATP1A1* knockdown. **(F)** A498 and 786-O cells scratching after *ATP1A1* knockdown. 0.1234(ns), 0.0332(*), 0.0021(**), 0.0002(***), <0.0001(****).

Analysis of the tumor immune microenvironment revealed that *ATP1A1* expression was significantly correlated with endothelial cells and naïve B cells. To explore the regulatory role of *ATP1A1* in renal cell carcinoma, four siRNA sequences targeting *ATP1A1* were designed to downregulate its expression. CCLE data shows that *ATP1A1* is highly expressed in the A498 and 786-O cell lines (**Figure 9A**). Subsequent qRT-PCR (**Figure 9B**) and western blot analysis (**Figure 9C**) confirmed effective knockdown, with si*ATP1A1*#2 and si*ATP1A1*#4 showing the highest silencing efficiency; these two sequences were selected for further functional experiments. CCK-8 assays (**Figure 9D**) demonstrated that *ATP1A1* knockdown significantly enhanced the proliferative capacity of renal carcinoma cells. Transwell migration assays (**Figure 9E**) and wound healing assays (**Figure 9F**) further showed that *ATP1A1* suppression markedly promoted cell invasion and migration compared to control groups. These findings highlight the potential of *ATP1A1* as a therapeutic target in the treatment of KIRC.

### 3.8 Molecular docking analysis of Emodinanthrone and ATP1A1

**Figure 10:**
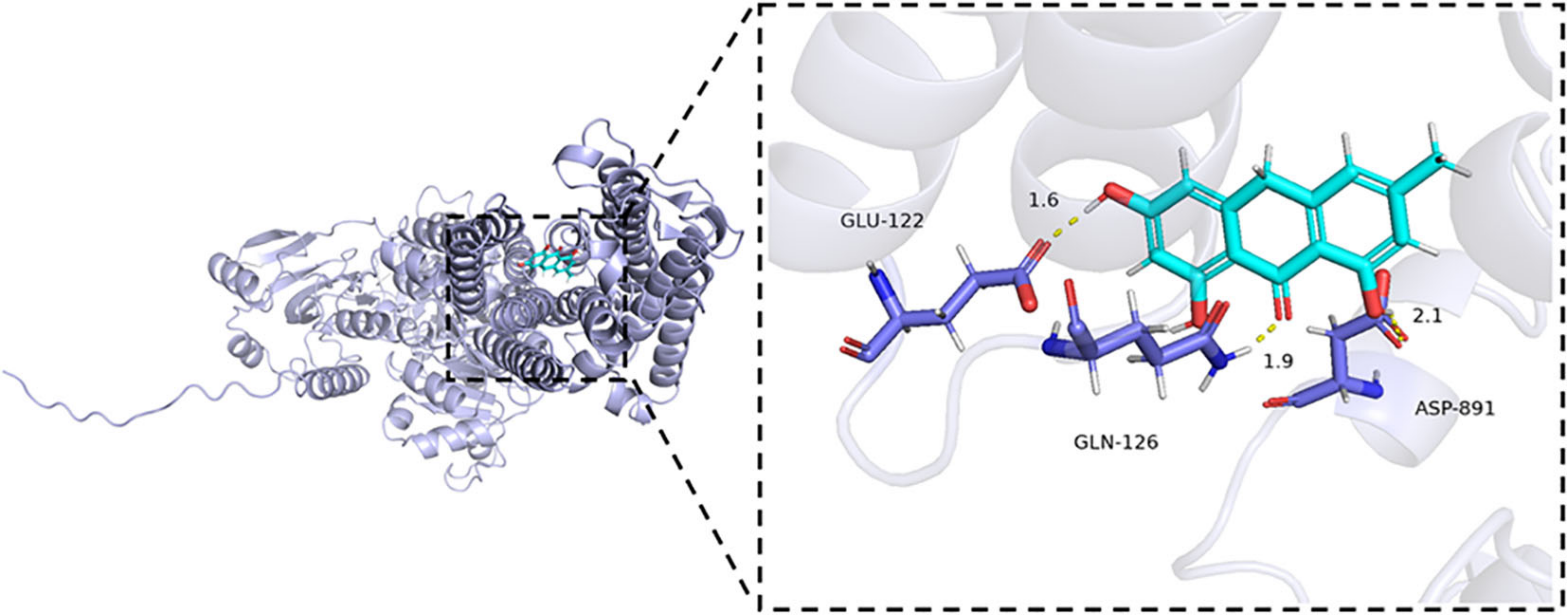
Molecular docking analysis of ATP1A1 and Emodinanthrone. A detailed 3D molecular docking model illustrating the binding affinity and interaction between ATP1A1 and Emodinanthrone. The protein is shown in blue, and the Emodinanthrone compound is depicted in cyan. Key residues are represented as sticks, with hydrogen bond interactions between amino acids and Emodinanthrone indicated by yellow dashed lines.

To begin the molecular docking analysis, we first prepared the compound library of traditional Chinese medicine and the protein structure using the methods described previously. High-throughput virtual screening was then conducted to identify compounds with promising interactions. Based on the docking scores, the top 20 compounds with the strongest binding energy (as shown in **Supplementary Table S2**) were selected for further analysis. A smaller binding energy value generally indicates stronger binding affinity, with values lower than -5 kcal/mol being considered favorable for binding. Among the top compounds, Emodinanthrone exhibited the best binding energy and was selected for a detailed 3D binding mode and interaction analysis. The docking results revealed a favorable binding energy of -6.800 kcal/mol between Emodinanthrone and ATP1A1. Notably, Emodinanthrone interacts with several key amino acids on ATP1A1, forming three hydrogen bonds with GLN-126, GLU-122, and ASP-891. These interactions are critical for stabilizing the protein-ligand complex and are likely to play a vital role in the compound’s biological activity. The visual analysis of these interactions is depicted in **Figure 10**.

### 3.9 Pan-cancer analysis of *ATP1A1* expression

**Figure 11:**
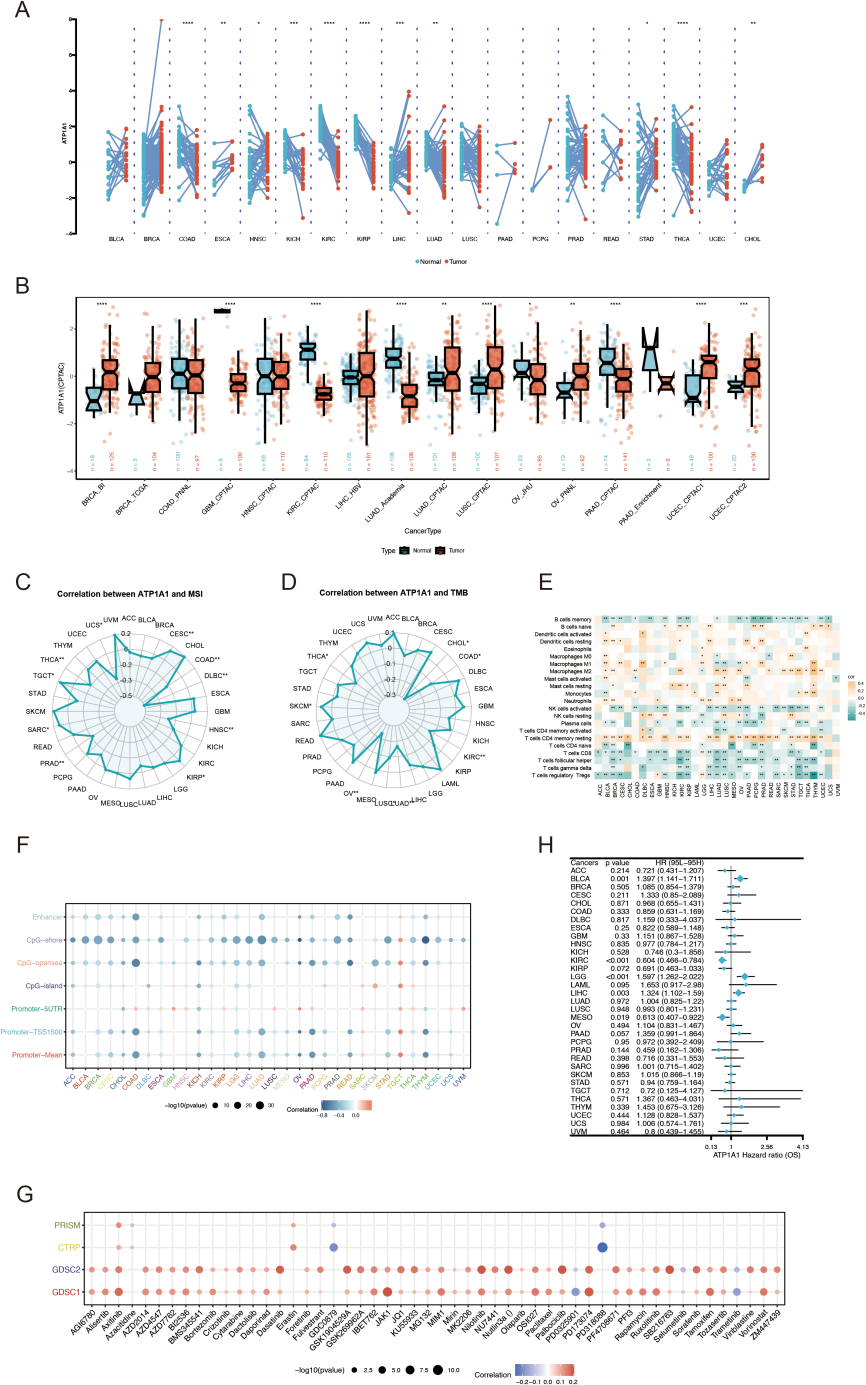
Pan-cancer analysis of *ATP1A1* expression, immune features, and genetic alterations. **(A)** ATP1A1 expression levels across TCGA tumor types and adjacent normal tissues. **(B)** Differential ATP1A1 protein levels (mass spectrometry) between cancer and normal tissues in the CPTAC database. **(C)** Correlation between ATP1A1 expression and MSI across TCGA datasets. **(D)** Association between ATP1A1 expression and TMB in TCGA datasets. **(E)** Heatmap showing the relationship between ATP1A1 expression and immune cell infiltration among various cancers. **(F)** Pearson correlation between ATP1A1 expression and immune-related genes in pan-cancer. **(G)** Spearman correlation between ATP1A1 expression and chemotherapy drug sensitivity across pan-cancer. **(H)** Pan-cancer Cox regression analysis of ATP1A1 as a prognostic factor across TCGA cancers. *p < 0.05; ***p < 0.001; ****p < 0.0001.

RNA sequencing data from the TCGA database to assess the expression of *ATP1A1* across multiple cancer types using the TCGAplot R package. The results indicated that *ATP1A1* was significantly overexpressed in ESCA, HNSC, LIHC, STAD, and CHOL, while its expression was notably lower in COAD, KIRP, KIRC, KICH, THCA, and LUAD (**Figure 11A**). Protein expression analysis revealed significant differences in ATP1A1 levels between tumor and normal tissues. Specifically, ATP1A1 protein expression was significantly reduced in the tumor samples of KIRC, GBM, LUAD, and PAAD compared to normal tissues (**Figure 11B**). Further, we examined the correlation between ATP1A1 expression and microsatellite instability (MSI). The radar chart showed a positive correlation of ATP1A1 with MSI in CESC, PRAD, SARC, and TGCT (**Figure 11C**). Similarly, analysis of the correlation between ATP1A1 expression and tumor mutational burden (TMB) revealed a positive association in SKCM (**Figure 11D**). To explore the relationship between *ATP1A1* and the immune microenvironment, we investigated the correlation between ATP1A1 expression and immune cell infiltration levels (**Figure 11E**). *ATP1A1* expression was negatively correlated with the infiltration of regulatory T cells (Tregs), follicular helper T cells, CD8+ T cells, CD4+ T cells, and memory B cells across most TCGA cancers, while it showed a positive correlation with macrophage infiltration in various cancers. Additionally, we examined the methylation patterns of *ATP1A1* across different cancer types and genetic loci (**Figure 11F**). As shown in **Figure 11G**, *ATP1A1* expression showed a positive correlation with sensitivity to several chemotherapy drugs in pan-cancer. Finally, to assess the potential clinical relevance of *ATP1A1*, univariate Cox regression analysis identified *ATP1A1* as a significant prognostic factor for OS in cancer patients (**Figure 11H**).

## 4 Discussion

KIRC, the most aggressive and immunogenic subtype of RCC, is characterized by high intratumoral heterogeneity, a strong propensity for early metastasis, and profound metabolic dysregulation within the TME, factors that collectively drive treatment resistance and disease recurrence (28, 29). Although the introduction of targeted therapies (e.g., pazopanib and sunitinib) and ICIs (e.g., nivolumab) has markedly improved survival outcomes for advanced-stage patients (30), the dynamic imbalance of metabolic byproducts such as lactate and ammonia within the TME may compromise therapeutic efficacy by promoting immune suppression and enabling tumor cells to evade cell death (31).

Emerging evidence highlights the intricate molecular mechanisms underpinning AICD and its critical role in tumor progression. Elevated ammonia levels have been shown to disrupt tricarboxylic acid (TCA) cycle flux by inducing glutamate dehydrogenase 2 (GDH2)-mediated aberrant reductive amination of α-ketoglutarate (α-KG), leading to simultaneous dysfunction of mitochondrial oxidative phosphorylation and glycolysis (32). This metabolic collapse is marked by inhibition of ATP synthase activity and excessive accumulation of reactive oxygen species (ROS), ultimately triggering mitochondrial membrane potential collapse and activation of caspase-dependent apoptotic pathways (33). Concurrently, ammonia-induced oxidative stress disrupts intracellular redox balance, activating a sustained unfolded protein response (UPR) through the PERK-eIF2α-ATF4 axis. This response upregulates CHOP, a pro-apoptotic transcription factor that simultaneously inhibits anti-apoptotic Bcl-2 family proteins (e.g., Mcl-1) and activates pro-apoptotic factors (e.g., Bim), thereby facilitating endoplasmic reticulum (ER) stress-mediated apoptosis (34–36). Importantly, ammonia exerts a concentration-dependent, dynamic effect on cellular homeostasis. At lower concentrations, it activates adaptive autophagy via the AMPK/mTOR pathway to maintain metabolic equilibrium (37); however, prolonged or excessive ammonia exposure leads to lysosomal dysfunction, impaired autophagic flux, and pathological accumulation of damaged organelles and misfolded proteins, leading to irreversible cellular injury (38). Despite advances in elucidating these mechanisms, the prognostic significance of AICD-related genes in KIRC and their associated regulatory networks has yet to be comprehensively explored through systematic bioinformatics approaches.

This study systematically elucidates, for the first time, the molecular features and clinical relevance of AICD-related genes in KIRC through comprehensive integration of multi-omics data. It further investigates their associations with the tumor microenvironment, immunotherapy responsiveness, potential targeted therapeutic strategies, and pan-cancer characteristics. Initially, we identified 228 DEARGs, among which *ANK3* exhibited the highest mutation frequency. Functional assessment indicated that these DEARGs are primarily responsible for key biological processes such as carboxylic acid metabolism, the HIF-1 signaling pathway, hypoxic responses, and cellular homeostasis, highlighting a potential mechanistic link between AICD, tumor metabolic reprogramming, and microenvironmental adaptation. The activation of the HIF-1 signaling pathway represents a pivotal mechanism by which tumors adapt to hypoxic stress. Li et al. demonstrated that HIF-1 signaling transcriptionally upregulates MRPL52, thereby regulating mitochondrial autophagy, ROS balance, and epithelial-mesenchymal transition (EMT), which are critical for hypoxia-driven metastatic progression in breast cancer (39). In our study, high-frequency mutations (56%) of the VHL gene observed in the HR patient group likely contribute to abnormal stabilization of HIF-α, resulting in persistent activation of its downstream targets (40, 41). This dysregulation may enhance glycolysis (the Warburg effect) (42) and upregulate enzymes associated with ammonia metabolism (e.g., glutaminase), thereby promoting excessive intracellular ammonia production. Furthermore, dysregulation of carboxylic acid metabolic pathways may exacerbate ammonia accumulation, establishing a “metabolism-hypoxia” positive feedback loop that perpetuates metabolic imbalance. Critically, ammonia-driven HIF-1 stabilization not only promotes glycolytic reprogramming (the Warburg effect) but may also directly suppress mitochondrial β-oxidation—a process central to HADH function—thereby exacerbating lipid metabolic dysfunction. Concurrently, plasminogen (PLG), a key regulator of proteolytic activity in the tumor microenvironment, may facilitate ammonia-mediated extracellular matrix remodeling through HIF-1-induced protease activation, further amplifying invasive potential. This disruption of metabolic homeostasis not only impairs cellular energy balance but may also influence epigenetic regulation, such as histone demethylation, through the depletion of key intermediates like α-ketoglutarate, ultimately resulting in cell death. Additionally, toxic ammonia accumulation can directly impair mitochondrial function, augment ROS production, and dysregulate redox enzyme activities, collectively amplifying oxidative stress (43). Accumulation of ROS leads to oxidative degradation of lipids, proteins, and DNA, resulting in lipid peroxidation, protein misfolding, and genomic instability (44). Moreover, ammonia has been shown to impair cellular antioxidant defenses by inhibiting the activity of key antioxidant enzymes (45), further compounding oxidative damage. This synergistic “oxidative-ammonia toxicity” may activate non-canonical forms of programmed cell death (e.g., necroptosis, ferroptosis), resulting in disruption of the plasma membrane and the release of intracellular components, thereby intensifying inflammatory responses within the tumor microenvironment.

Further analyses utilizing spatial transcriptomics and scRNA-seq revealed distinct, cell type-specific expression patterns of AICD-associated genes, with vascular endothelial cells exhibiting markedly higher activation levels compared to other cellular subpopulations. This observation is likely due to the dual functional roles of endothelial cells within the tumor microenvironment: as principal regulators of angiogenesis, endothelial cells likely modulate tumor vascularization and nutrient delivery through pathways linked to ammonia metabolism (46). Mechanistically, ammonia has been shown to enhance endothelial angiogenic capacity via activation of mTORC1 signaling, thereby promoting amino acid biosynthesis (10), while an acidic microenvironment mitigates ammonia toxicity to preserve vascular homeostasis and permeability (47). Conversely, the elevated activation of AICD-related genes in endothelial cells may also suggest that dysregulated ammonia metabolism contributes to endothelial dysfunction, potentially fostering abnormal tumor vasculature and remodeling the immune microenvironment. Notably, the weak correlation observed between CD8^+^ T cells and plasma cells may reflect mechanisms of tumor immune evasion, possibly mediated by ammonia-driven immunosuppressive processes. These findings propose new therapeutic avenues for KIRC, including the targeting of high-frequency mutated genes (e.g., ANK3) or endothelial-specific metabolic vulnerabilities. Furthermore, they underscore the central role of ammonia metabolism as a critical regulator of tumor microenvironmental dynamics. Future studies should focus on experimentally validating the functional impacts of ANK3 mutations within endothelial cells and further elucidating the mechanistic interplay between ammonia metabolism and IC regulation.

Based on the five core genes (*FOXM1*, *ANK3*, *ATP1A1*, *HADH*, and *PLG*) identified from the TCGA-KIRC cohort, a prognostic risk scoring model was successfully established. This model demonstrated robust survival stratification and strong clinical applicability across both external and internal validation cohorts. According to the relative risk ratios, *FOXM1* was identified as a risk factor, whereas *ANK3*, *ATP1A1*, *HADH*, and *PLG* were classified as protective factors. *FOXM1*, a critical regulator of the cell cycle, governs the transitions between the S and G2/M phases (48). Extensive research implicates *FOXM1* in the progression of various malignancies (49–53). Studies have demonstrated that *FOXM1* facilitates G2/M phase transition by directly regulating CDC2 phosphorylation, thereby accelerating mitosis (54). *ANK3*, which encodes an immunospecific member of the ankyrin family, is widely expressed across nephron segments and functions as a tumor suppressor in multiple cancer types (55, 56). Notably, a study by Yunshan Zhu et al. demonstrated that *ANK3* downregulation is associated with cisplatin resistance in ovarian carcinoma (57). *ATP1A1*, the core subunit of the sodium-potassium pump, may contribute to ammonia-induced cell death and tumor immune evasion by disrupting ionic homeostasis and reprogramming ammonia metabolism. Although ATP1A1 exerts oncogenic functions via the ERK5 pathway in colorectal cancer (58), it prolongs survival in renal carcinoma by suppressing Raf/MEK/ERK signaling (59), consistent with our *in vitro* findings, where ATP1A1 knockdown enhanced proliferation, migration, and invasion in renal cancer cells. This functional dichotomy is likely attributable to microenvironmental differences in ammonia metabolism. Ammonia toxicity has been shown to directly induce effector T-cell exhaustion through the collapse of mitochondrial membrane potential and lysosomal damage (60). ATP1A1 dysregulation further exacerbates intracellular sodium-potassium gradient disruption, compromising pH balance (61). Single-cell sequencing data reveal endothelial-specific overexpression of ATP1A1, suggesting a role in maintaining vascular barrier integrity and modulating local ammonia clearance (62). This may indirectly suppress CD8^+^ T-cell activity, contributing to a vicious cycle of “ammonia accumulation–immune exhaustion. Mechanistically, ATP1A1 triggers an immunosuppressive tumor microenvironment through multidimensional crosstalk with the ammonia metabolism-immune checkpoint axis. In melanoma, ATP1A1 forms a complex with Cav-1 to activate Src/AKT signaling (63), promoting therapeutic resistance and T-cell suppression. In lung cancer, ATP1A1 stabilizes PD-L1 expression (64) and regulates other immune checkpoints such as CEACAM-1 and B7-H3 (65), thereby inhibiting antigen presentation. ATP1A1 also governs macrophage polarization by interacting with Lyn kinase to promote oxLDL-CD36-mediated lipid overload and M2 polarization (66), implicating metabolic reprogramming in immunosuppressive cell recruitment. Clinically, ATP1A1 expression correlates with tumor T stage and venous invasion in gastric cancer (67) and holds prognostic significance in ovarian cancer (68). Its pan-cancer immunomodulatory effects may be mediated through STAT1-IDO1 signaling (60) or NF-κB-driven inflammatory pathways (65), ultimately promoting Treg infiltration and immune checkpoint expression (64). Targeting the ATP1A1-ammonia axis represents a promising therapeutic strategy for overcoming treatment resistance. Bufalin disrupts the ATP1A1-Cav-1 complex, reversing drug resistance in melanoma (63), while cardiac glycosides inhibit STAT1-mediated IDO1 expression (60), demonstrating synergy with immune checkpoint inhibitors. ATP1A1 knockdown has also been shown to reverse ammonia-induced mitochondrial dysfunction in T cells (60). Furthermore, ammonia-scavenging agents, such as urea cycle activators, may help reprogram the metabolic-immune landscape to enhance therapeutic efficacy (69). Future research should focus on spatial metabolomics to map ammonia distribution in ATP1A1-deficient tumors, the development of isoform-selective inhibitors (e.g., α1-targeting agents), and the exploration of combination therapies involving epigenetic modulators or macrophage metabolic reprogramming to overcome ammonia-induced immunotherapy resistance. *HADH*, which encodes 3-hydroxyacyl-CoA dehydrogenase, plays a pivotal role in mitochondrial fatty acid β-oxidation (70). In colorectal cancer, *HADH* has been implicated in non-canonical Wnt signaling, regulating tumor proliferation and metabolic activity via the Wnt5a/b-Ror2/Dvl2-ATF2/4 axis (71). Plasminogen (*PLG*), a glycoprotein synthesized in the liver, is activated by plasminogen activators and is critically involved in proteolytic processes within the tumor microenvironment (72). Elevated expression of plasminogen activators has been linked to enhanced invasion and migration in pancreatic ductal adenocarcinoma cells (73). Additionally, *PLG* has been implicated in the development and progression of several malignancies, including lung cancer (74), breast cancer (75), colorectal cancer (76), and meningiomas (77).

Further analyses revealed extensive immune cell infiltration within the TMEs across different risk subgroups. Notably, KIRC displays distinct TME characteristics and clinical behavior compared to most other solid tumors (78). While in many malignancies high CD8^+^ T cell infiltration correlates with favorable prognosis, KIRC exhibits a paradoxical association where elevated CD8^+^ T cell presence is linked to poorer clinical outcomes (79). This discrepancy may be attributed to the concurrent enrichment of immunosuppressive cell types, such as Tregs and TAMs, which can mitigate the antitumor efficacy of cytotoxic T cells (80). In addition, gene-specific immune associations were observed: *FOXM1* showed significant positive correlations with macrophage infiltration, while *ATP1A1* was notably associated with B cell populations. Mechanistic insights from Rong Xu et al. (81)demonstrated that PLK1-mediated phosphorylation activates *FOXM1* to promote pro-tumorigenic macrophage polarization by regulating inflammatory cytokine production, while simultaneously upregulating PD-L1 expression, thereby enhancing immune evasion and metastatic potential. Moreover, we found that in the high-risk group of KIRC, the negative correlation between CD8^+^ T cell infiltration and poor prognosis is mainly due to the functional exhaustion of CD8^+^ T cells rather than abnormal numbers: in the high-risk group, the expression of the immune checkpoint molecule PDCD1 (PD-1) is significantly upregulated, and the TIDE score is elevated, indicating that CD8^+^ T cells are in a state of functional impairment due to continuous immunosuppressive signals. Although they are infiltrated, they cannot effectively exert anti-tumor effects. At the same time, metabolic disorders such as ammonia accumulation and hypoxia in the high-risk group further damage CD8^+^ T cell activity by activating the HIF-1 signaling pathway, leading to a state of “presence but paralysis” of function, which results in the negative correlation, and this contradiction does not depend on the co-infiltration of Tregs (82). Subsequently, we analyzed the expression differences of immune checkpoint molecules in patients of different risk groups. In contrast, the expression levels of CD274 (PD-L1), HAVCR2 (TIM-3), and HHLA2 in the high-risk group were significantly lower than those in the low-risk group, while the expression of PDCD1 (PD-1), TMIGD2, and TIGIT was significantly higher. The downregulation of PD-L1 may reflect the tumor’s active avoidance of immune recognition by reducing immune exposure and antigen presentation. In contrast, the high expression of PD-1 and TIGIT indicates that immune cells are under prolonged antigen stimulation, exhibiting a state of deep exhaustion (83, 84). However, in this case, PD-1 is upregulated in the high-risk group, while PD-L1 is downregulated, suggesting a possible alteration in the immune evasion mechanism or a shift of the tumor toward a PD-L1-independent inhibitory pathway. Moreover, TIDE analysis indicated that HR patients exhibited significantly higher scores, suggesting an elevated propensity for tumor immune escape and potentially reduced responsiveness to IC blockade therapies. At the genomic level, HR patients demonstrated significantly increased mutation frequencies in *VHL* (56% vs. 45%) and *PBRM1* (46% vs. 39%). Loss of *VHL* function activates hypoxia-inducible factor (HIF) signaling by stabilizing HIF-α proteins, thereby promoting tumor angiogenesis, metabolic reprogramming, and ammonia accumulation (85, 86). Concurrently, *PBRM1* mutations, by disrupting SWI/SNF chromatin remodeling complex activity, may alter chromatin accessibility and impact the transcriptional regulation of ammonia metabolism-related genes (87). The synergistic effects of these mutations contribute to enhanced tumor heterogeneity, metabolic dysregulation, and adverse clinical outcomes.

Furthermore, the elevated TMB and increased MSI observed in the HR group indicate a heightened level of genomic instability, potentially enhancing tumor immunogenicity through increased neoantigen generation (88). However, the presence of a profoundly immunosuppressive tumor microenvironment (TME) in these patients may attenuate the anticipated immunogenic benefits, suggesting the potential therapeutic advantage of combinatorial strategies that concurrently target DNA repair mechanisms (e.g., PARP inhibitors) and IC pathways (89). Copy number variation (CNV) analysis revealed significantly greater genomic instability in the HR subgroup compared to the LR cohort. Notably, lower ATP1A1 expression was positively correlated with enhanced sensitivity to certain chemotherapeutic agents. These data imply that ATP1A1 expression levels could serve as a predictive biomarker for chemotherapy response, potentially through its regulatory effects on ion transport dynamics and membrane integrity. Moreover, distinct CNV profiles between risk groups may modulate drug target gene expression and key oncogenic signaling pathways, offering novel insights for the development of personalized therapeutic approaches. The implications of these differential CNV landscapes in precision oncology warrant further investigation.

Natural compounds represent a promising reservoir for cancer chemoprevention and therapy. In this study, molecular docking analyses based on the structural configuration of ATP1A1 identified Emodinanthrone as a promising candidate compound. Docking results demonstrated favorable binding affinity and robust molecular interactions between ATP1A1 and Emodinanthrone. While this computational prediction suggests strong binding potential, functional inhibition of ATP1A1 requires further validation through wet laboratory experiments. Previous research has established that Emodinanthrone functions as a biosynthetic precursor for hypericin (90), a photodynamic therapeutic agent with demonstrated efficacy across various malignancies (91–94). These findings suggest that Emodinanthrone holds potential for improving clinical outcomes in cancer treatment. However, its direct therapeutic application in KIRC remains to be fully validated, necessitating further preclinical and clinical investigations to assess its efficacy and safety profiles. Additionally, elucidating the mechanistic interplay between Emodinanthrone’s binding to ATP1A1 and its downstream pharmacodynamic effects will be essential for determining its clinical relevance in oncological therapy.

In the pan-cancer analysis of ATP1A1, we also discovered the heterogeneity of ATP1A1, showing completely opposite effects in different tumor types. For example, the expression of ATP1A1 is significantly upregulated in liver hepatocellular carcinoma (LIHC), exerting a carcinogenic effect. Pradeep Kumar Rajan et al. demonstrated that physiological ATP1A1 expression in HCC cells exerts antitumor effects via epigenetic regulation and enhancement of autophagy, suggesting that ATP1A1 dysregulation may contribute to metabolic reprogramming and tumor progression. Further characterization of the immune microenvironment across malignancies revealed distinct immunological patterns in KIRC, typified by elevated infiltration of M2-polarized macrophages, resting memory CD4+ T cells, and mast cells, alongside reduced infiltration of Tregs and follicular helper T cells (Tfh). Flow cytometry-based profiling of tumor-infiltrating lymphocytes across cancers confirmed substantial infiltration of both CD8+ and CD4+ T cells in KIRC (95). Notably, in head and neck cancers, high infiltration of M2 macrophages and resting memory CD4+ T cells is associated with poor clinical outcomes (96), whereas Treg-mediated suppression of effector T cell function can dampen antitumor immunity (97), highlighting the complex and heterogeneous immunobiology across different tumor types. Multivariate survival analyses established *ATP1A1* as a significant prognostic factor, highlighting the necessity for functional verification experiments to further elucidate its therapeutic potential in KIRC.

Although this research demonstrated the prognostic utility of the AICD scoring system through multi-cohort validation and revealed its associations with immune microenvironmental features and genomic alterations, several limitations must be acknowledged. First, the analyses were predominantly based on retrospective datasets such as TCGA and E-MTAB-1980. Although cross-validation across independent cohorts minimized potential biases, the applicability of these findings to real-world, prospective clinical populations remains to be confirmed. Second, although bioinformatic analyses implicated AICD-related genes in pathways (e.g., HIF-1 signaling and oxidative stress-mediated cell death), the functional roles and regulatory networks of these genes have yet to be validated through experimental approaches, such as patient-derived organoid models or *in vivo* systems. Third, immune microenvironment characterization relied heavily on computational deconvolution algorithms and spatial transcriptomic datasets with limited sample sizes (e.g., GSE139555 comprising only three patients), which may inadequately represent the full heterogeneity of KIRC. Future research should aim to incorporate larger sample sizes, multi-omics integration, and experimental validation to strengthen the biological and clinical relevance of these findings.

This study presents a comprehensive investigation into the prognostic significance of AICD-related genes in KIRC. A prognostic model centered on *ATP1A1* exhibited strong predictive performance in external validation cohorts. Notable differences were observed between risk groups in terms of immune microenvironment composition, genomic instability, and immune checkpoint expression. Functional assays confirmed that *ATP1A1* knockdown significantly promoted tumor cell proliferation, migration, and invasion. Critically, molecular docking identified the natural compound Emodinanthrone as a high-affinity ligand for ATP1A1, suggesting its potential as a novel therapeutic agent for targeting the ammonia-immunometabolic axis in KIRC. Additionally, pan-cancer analyses revealed *ATP1A1*’s involvement in metabolic regulation, therapeutic target, and immune cell infiltration, offering mechanistic insights into KIRC pathogenesis. Collectively, these findings identify *ATP1A1* as a promising prognostic biomarker and therapeutic target, paving the way for personalized treatment strategies in KIRC.

## Data Availability

The datasets presented in this study can be found in online repositories, or are available from the corresponding author upon reasonable request.
