# Supplementary figures and images for: Unveiling ammonia-induced cell death: a new frontier in clear cell renal cell carcinoma prognosis
Source: Front Immunol. 2025 Jul 31;16:1636977. doi: 10.3389/fimmu.2025.1636977 (PMC12350399; doi:10.3389/fimmu.2025.1636977)

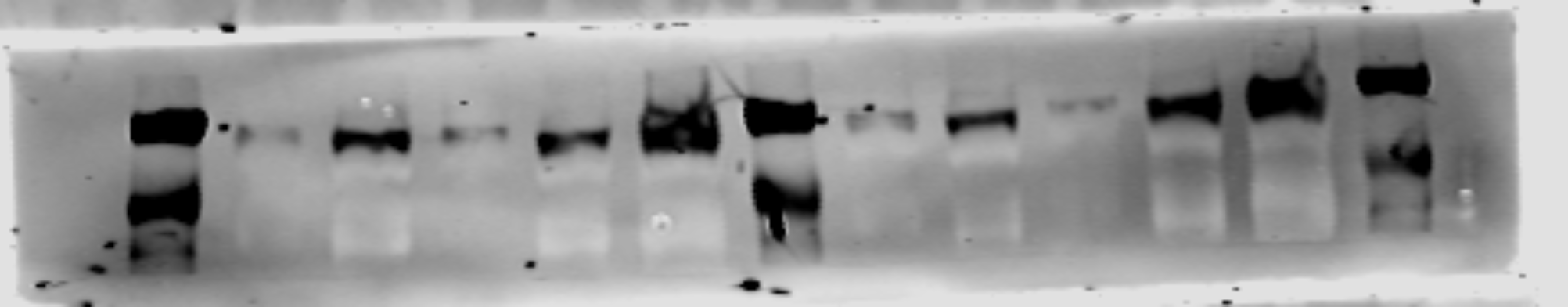

Supplement: Supplementary file 1 [file Image1.tif]

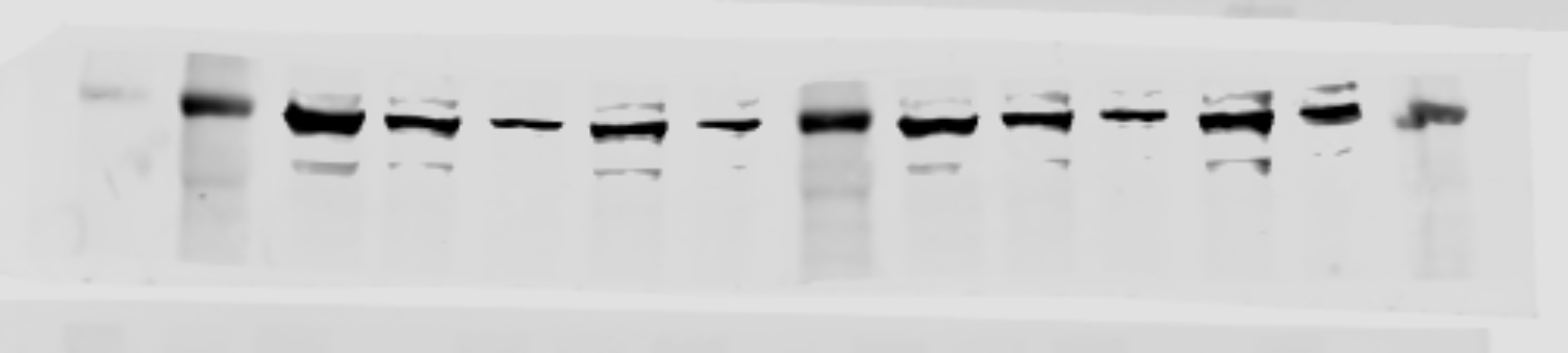

Supplement: Supplementary file 2 [file Image2.tif]

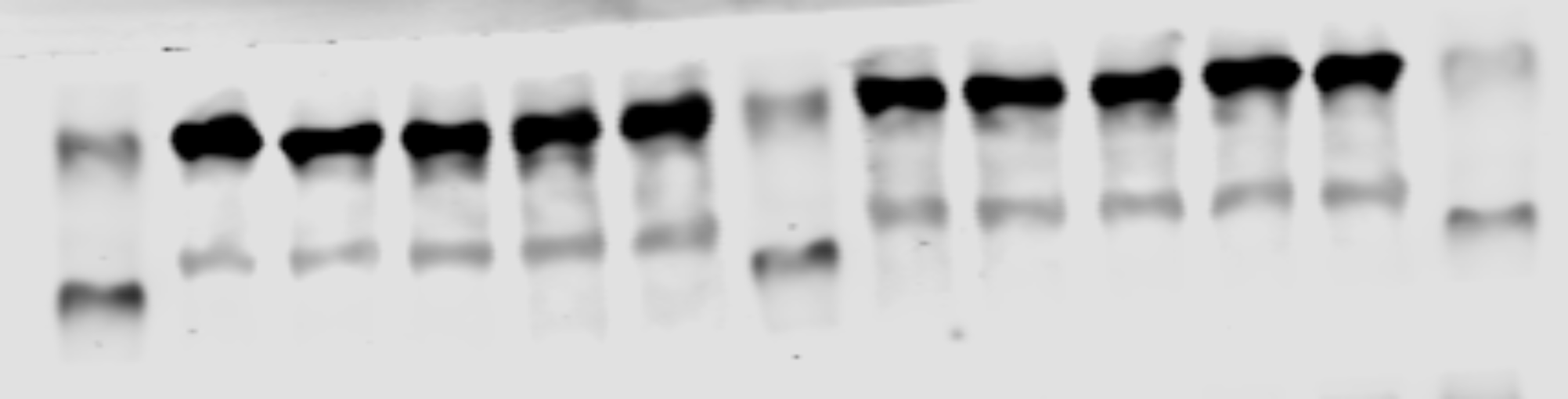

Supplement: Supplementary file 3 [file Image3.tif]

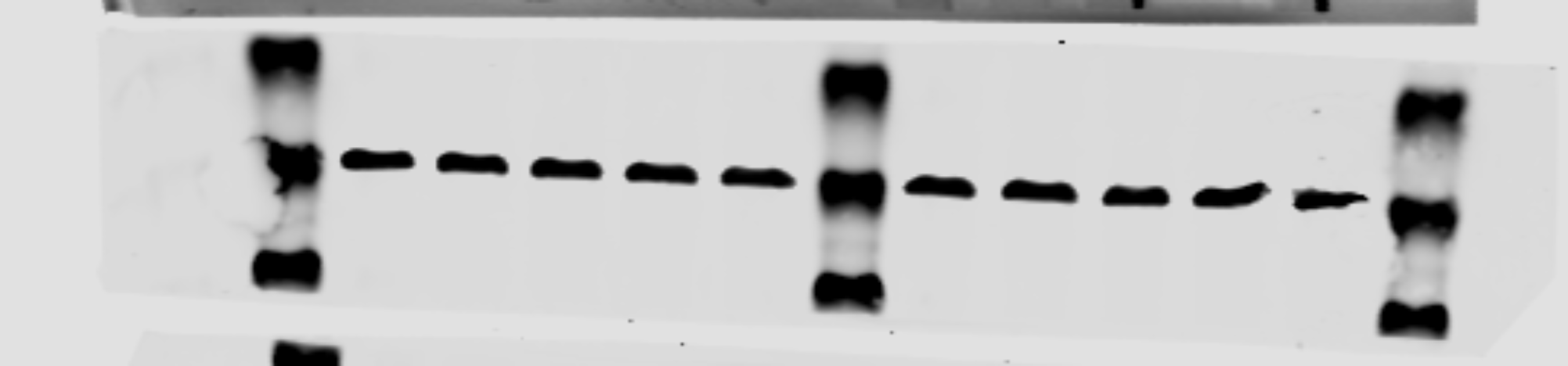

Supplement: Supplementary file 4 [file Image4.tif]
